# Interhemispheric EEG coherence as a candidate biomarker in gambling disorder: evidence of frontal hyperconnectivity and posterior disconnectivity

**DOI:** 10.3389/fnins.2025.1687112

**Published:** 2025-10-24

**Authors:** Eda Yılmazer, Metin Çinaroğlu, Selami Varol Ülker, Sultan Tarlacı

**Affiliations:** ^1^Psychology Department, Faculty of Social Science, Beykoz University, Istanbul, Türkiye; ^2^Psychology Department, Faculty of Administrative and Social Science, Istanbul Nişantaşi University, Istanbul, Türkiye; ^3^Psychology Department, Faculty of Humanities and Social Science, Üsküdar University, Istanbul, Türkiye; ^4^Neurology, Medical School, Üsküdar University, Istanbul, Türkiye

**Keywords:** gambling disorder, qEEG coherence, biomarker, interhemispheric connectivity, orbitofrontal hyperconnectivity, addiction neuroscience

## Abstract

**Background:**

Gambling Disorder (GD) is a behavioral addiction marked by impaired decision-making and poor impulse control. We investigated whether resting-state interhemispheric quantitative EEG (qEEG) coherence—a measure of functional connectivity between homologous cortical regions—could serve as a biomarker of GD.

**Methods:**

Twenty-nine male patients with GD and 45 healthy male controls underwent resting-state qEEG recording. Coherence was computed for homologous electrode pairs across delta, theta, alpha, and beta bands. Group differences were analyzed using independent-samples *t*-tests; associations with disorder duration were assessed via age-controlled partial correlations.

**Results:**

Consistent with our hypothesis, GD participants exhibited frontal pole hypercoherence (Fp1–Fp2) across delta, theta, and beta bands, which is likely influenced by prefrontal/orbitofrontal generators. In contrast, GD showed hypocoherence in temporal (T3–T4, T5–T6), central (C3–C4), and parietal (P3–P4) regions across these frequencies. Greater disorder duration was associated with lower beta coherence at F3–F4 and Fp1–Fp2, and higher delta coherence at O1–O2.

**Conclusions:**

These findings reveal a dual pattern of interhemispheric connectivity disruption in GD—hypercoherence at frontal pole sites and hypocoherence in sensorimotor and attentional posterior networks—supporting theoretical models of addiction neurocircuitry. Resting-state qEEG coherence holds promise as a clinically relevant biomarker for GD and may inform the development of neuromodulatory interventions aimed at network rebalancing.

## 1 Introduction

### 1.1 EEG and coherence: a window into functional connectivity

Electroencephalography (EEG) provides a noninvasive measure of brain electrical activity with millisecond resolution, capturing oscillatory dynamics across different frequency bands (Müller-Putz, 2020). One important metric derived from EEG is coherence, which quantifies the degree of phase synchrony between signals from two recording sites in the frequency domain (Guevara and Corsi-Cabrera, 1996). In simpler terms, a high coherence implies that two brain regions are oscillating in near-unison at a particular frequency, whereas low coherence suggests their activity is uncorrelated or independent (Leocani and Comi, 1999). This measure has long been used to assess functional coupling in the brain. In fact, since the 1960s coherence analysis has been applied to EEG data to determine if two sensors (and by extension, the cortical areas beneath them) have “similar neuronal oscillatory activity” (Shaw, 1981). Because coherent EEG activity reflects synchronized neuronal oscillations, it is interpreted as an index of functional connectivity between neural populations (Ganzetti and Mantini, 2013). In neurophysiology, such synchrony is thought to underlie communication within and between brain networks, as neurons firing in phase can more effectively transmit information (Michels et al., 2013). Thus, EEG coherence provides a window into how different brain regions functionally interact in resting-state or during tasks (Bowyer, 2016). Notably, coherence is frequency-specific—connectivity may be strong in one band (e.g., theta) but weak in another (e.g., beta), offering a nuanced view of network coupling across the spectrum (González et al., 2020).

Beyond its technical definition, EEG coherence holds significant neurobiological meaning. Synchronized oscillations between regions reflect shared information processing or functional integration, while desynchronized activity may reflect functional segregation (Leocani et al., 1997). High coherence between two EEG signals therefore suggests the corresponding brain areas are part of the same functional network, dynamically coupling their activity. Conversely, abnormally low coherence could indicate a breakdown in communication pathways or dysregulation of network connectivity (Knyazeva and Innocenti, 2001). Because brain function relies on both specialized regional processing and integrated network communication, coherence metrics are valuable for characterizing the balance between integration and segregation in the brain's resting-state activity (Tononi et al., 1998). In summary, EEG coherence serves as a quantitative marker of brain functional connectivity, capturing the alignment of oscillatory phase between regions over time. It has been widely applied in cognitive and clinical neuroscience as a tool to map brain networks and understand how synchrony (or lack thereof) relates to behavioral and clinical phenomena.

### 1.2 EEG coherence as a biomarker in psychiatric and addictive disorders

Given its sensitivity to network synchrony, EEG coherence has been explored as a potential biomarker of neuropathology in various psychiatric and neurological conditions (Yun, 2024). Abnormal coherence—whether reduced (hypocoherence) or heightened (hypercoherence) compared to healthy norms—can signal disruptions in the brain's functional connectivity, which may underlie symptoms or cognitive deficits (Markovska-Simoska et al., 2018). For example, many disorders characterized by cognitive impairment or dysconnectivity show lowered coherence in certain frequency bands, reflecting weaker functional integration. Patients with bipolar disorder have been reported to exhibit diminished long-range coherence between frontal and temporal regions, indicating a reduction in network coupling (Özerdem et al., 2011). Similarly, Alzheimer's disease is associated with decreases in alpha- and beta-band coherence between distant cortical areas during resting state, consistent with a general breakdown of large-scale cortical networks in dementia (Locatelli et al., 1998). Schizophrenia, a prototypical dysconnection syndrome, has also been linked to reduced coherence in beta frequencies, suggesting impaired high-frequency synchrony in neural circuits (Yeragani et al., 2006). These hypocoherence findings align with the idea that certain pathologies involve a loss of efficient long-range communication (e.g., weakened frontoparietal or interhemispheric coordination).

On the other hand, some conditions show excessive or aberrant increases in coherence. In such cases, hypercoherence may reflect overly synchronized activity or reduced differentiation between regions—a potential sign of network inflexibility or abnormal excitation (Stam et al., 2023). For instance, one magnetoencephalography (MEG) study found schizophrenia patients to have increased coherence across a broad 3–50 Hz range in medial prefrontal and anterior cingulate regions compared to controls (Hinkley et al., 2010). This suggests that, in addition to the selective hypoconnectivity at higher frequencies, schizophrenia can involve aberrant hyper-synchronization in certain circuits (possibly related to positive symptoms or compensatory mechanisms). In the realm of addiction neuroscience, emerging evidence also points to coherence abnormalities. Chronic methamphetamine users in early abstinence showed significantly lower coherence in slow-wave bands (delta, theta) between certain regions (e.g., parietal cortex), indicative of compromised regional integration after stimulant neurotoxicity (Shafiee-Kandjani et al., 2020). By contrast, hypercoherence has been observed in some behavioral addictions: for example, individuals with Internet Gaming Disorder (IGD) demonstrated elevated resting-state coherence relative to both healthy controls and even alcohol use disorder patients (Park et al., 2018). Such high-frequency hypercoherence in a behavioral addiction has been interpreted as a marker of pathological hyperexcitability and over-engagement of certain networks. Taken together, these findings underscore that coherence deviations in either direction (too low or too high) can serve as network-level biomarkers of pathology. Different frequency bands may be differentially affected—for example, frontal theta coherence might be altered in disorders of executive function (Basharpoor et al., 2021), while diffuse alpha coherence reductions may accompany degenerative conditions (Nimmrich et al., 2015). Importantly, coherence changes have been linked to clinical features: for instance, in nicotine addiction, heightened low-theta coherence in frontoparietal networks during smoking cue exposure correlates with craving levels, suggesting a neurophysiological signature of cue-induced desire (Bu et al., 2019). Thus, assessing EEG coherence across delta, theta, alpha, beta bands provides a rich characterization of brain functional connectivity, and abnormalities in these measures are increasingly recognized in psychiatry and addiction research as potential biomarkers of dysfunctional neural circuitry. Recent EEG coherence studies in behavioral addictions further highlight the relevance of network-level disturbances. For example, Park et al. (2017) reported increased intrahemispheric gamma coherence in IGD, particularly in fronto-central regions, while Taigibova and Rabadanova (2022) found divergent coherence profiles in Internet vs. game addicts, with reduced frontal but heightened occipital coupling in the former and widespread frontal/temporal hypercoherence in the latter. These findings underscore that coherence alterations are not unique to substance addictions but also extend to behavioral addictions, motivating investigation of GD.

### 1.3 Resting-state EEG findings in gambling disorder

GD is a behavioral addiction characterized by persistent and maladaptive gambling behavior, recognized in DSM-5 alongside substance use disorders (Potenza et al., 2019). It affects approximately 0.4%−1.0% of the population (Stevens et al., 2021) and is frequently comorbid with substance addictions (Rash et al., 2016), mood disorders (Sharma and Weinstein, 2025), and Attention-Deficit/Hyperactivity Disorder [ADHD, (Karaca et al., 2017)]. Given its phenotypic overlap with other addictions (e.g., loss of control, impulsivity, craving), one might expect neurophysiological commonalities as well. Indeed, although research on GD's neural correlates is relatively nascent, early EEG studies suggest that gamblers have atypical resting brain activity patterns (Lee et al., 2017). Notably, pathological gamblers show dysfunctional EEG activity in frontoparietal regions compared to healthy individuals (Quintero, 2016). This observation aligns with neuroimaging studies pointing to altered function in prefrontal and parietal cortical areas (involved in decision-making and impulse control) in GD (Koehler et al., 2013). qEEG analyses of power spectra indicate that GD and related impulsive populations often exhibit increases in slow-wave activity (delta and theta bands) alongside alterations in faster rhythms (Kim et al., 2017). For example, a systematic review reported that individuals with problematic gambling tend to have elevated resting delta and theta power, coupled with atypical beta activity (Burleigh et al., 2020). These patterns mirror findings in substance use disorders and other impulse-control disorders, where increased power in low-frequency bands is thought to reflect low cortical arousal or an imbalanced reward system, and changes in beta power may reflect hyperarousal or executive dysregulation (Liu et al., 2022). In line with this, an increased theta–beta power ratio in resting EEG has been associated with riskier decision-making on the Iowa Gambling Task, a behavioral tendency relevant to gambling pathology (Massar et al., 2014). Additionally, asymmetries in frontal beta power have been linked to the frequency of risky choices (Neal and Gable, 2019), suggesting that not only the magnitude of power in certain bands but also its lateralization may carry significance in GD. While some inconsistencies exist across studies (likely due to heterogeneity in samples and methods), the overall picture indicates that GD is marked by deviations in baseline oscillatory activity—particularly, a bias toward slower frequencies (delta/theta) and, in some studies, atypical beta activity. These alterations have been tentatively interpreted as reflecting reduced cortical inhibition or heightened excitability related to craving, although the evidence remains preliminary and requires further confirmation.

Critically, one aspect that has received little attention in gambling disorder to date is EEG coherence, especially *interhemispheric* coherence. Most prior EEG studies in GD have focused on spectral power or event-related potentials (Simkute et al., 2024), which are sensitive to local cortical activity or time-locked responses, but they do not directly measure the coordinated interaction between brain regions. Functional connectivity in GD remains understudied, despite evidence from other modalities (e.g., fMRI) that addictive disorders involve network-level alterations (van Timmeren et al., 2018). Coherence analysis could fill this gap by revealing how synchronized or connected different brain regions are in resting-state for GD patients. In particular, interhemispheric coherence—the coherence between homologous electrodes in the left and right hemispheres—is of high interest. The degree of synchrony between the two hemispheres may reflect the integrity of transcallosal connections and the balance of bilateral neural processing. If gambling disorder involves atypical lateralization or impaired cross-hemisphere communication (as some neuropsychological studies imply), it might manifest as abnormal coherence between left-right pairs of electrodes.

To set the stage for anatomical interpretation of interhemispheric coherence, it is useful to note which brain areas correspond to the EEG electrode sites analyzed. In the standard 10–20 system, each scalp electrode can be approximated to overlie certain Brodmann areas (BAs) of the cortex. For example, the frontopolar electrodes Fp1–Fp2 are located over the left and right orbitofrontal cortex and frontal pole—roughly corresponding to Brodmann areas 10 and 11 in the anterior prefrontal region (Brodmann, 2006). Coherence between Fp1 and Fp2 thus reflects the functional coupling of the orbitofrontal cortices bilaterally, regions implicated in reward evaluation and impulse control [highly relevant to gambling behavior (Amarante and Laubach, 2021)]. Similarly, electrodes T3–T4 (also labeled as T7–T8 in the extended 10–10 system) sit over the left and right lateral temporal lobes. These positions encompass parts of the superior and middle temporal gyri, including auditory association areas and regions involved in language and emotion [approximated by BAs 21, 22, and 42 (Pitts et al., 2020)]. Coherence between T3 and T4 indexes the synchronization of activity between the left and right temporal cortices. By examining such interhemispheric pairs across the scalp (frontal, central, parietal, etc.), one can infer whether GD is associated with selective connectivity disturbances in particular cortical networks. For instance, reduced coherence at frontal pairs might suggest impaired frontal executive network integration between hemispheres, whereas altered temporal coherence could point to imbalances in auditory-limbic circuits. It should be noted that scalp EEG coherence is an indirect reflection of cortical interactions (volume conduction and reference choices can influence coherence), but when interpreted with anatomical awareness, it can provide meaningful clues about which brain regions' communication is disrupted.

### 1.4 Rationale and aims of the present study

Resting-state EEG research indicates that gambling disorder involves abnormal brain activity, yet the connectivity aspect of these abnormalities remains unclear. EEG coherence is a promising approach to quantify such connectivity, as it captures the synchronous activity between brain regions that could underlie the addictive and impulsive traits of GD. Both hypercoherence and hypocoherence could plausibly characterize GD: for example, excessive coherence might reflect rigid or over-engaged reward circuits, while deficient coherence might reflect a failure of executive control networks to coordinate effectively. To date, however, to the best of our knowledge, no comprehensive study has examined interhemispheric EEG coherence across multiple frequency bands in GD. Addressing this gap is crucial for a more complete neurophysiological model of GD. The present study was therefore designed to investigate resting-state interhemispheric EEG coherence in individuals with GD, across the delta (~1–4 Hz), theta (~4–8 Hz), alpha (~8–12 Hz), and beta (~12–25 Hz) frequency bands. By comparing GD patients to healthy control participants, we aim to determine whether GD is associated with significant coherence abnormalities in any of these bands. Our analysis focuses on coherence between homologous left-right electrode pairs (spanning frontal, temporal, central, and parietal regions) to specifically probe interhemispheric functional connectivity.

Building on prior findings in behavioral addictions, we hypothesized a dual pattern of abnormalities. Increases in frontal interhemispheric coherence were expected in delta, theta, and beta bands, reflecting hyper-integration of reward and executive circuits (Park et al., 2018). At the same time, we predicted reduced coherence in posterior regions (temporal, parietal, occipital) in alpha and beta bands, reflecting weakened sensory-attentional integration. These band- and region-specific expectations move beyond general assumptions about “slow-wave abnormalities” and ground our predictions in prior evidence from IGD alcohol dependence, and related addictions. By evaluating these possibilities, our goal is to elucidate whether measurable connectivity disruptions underlie the EEG differences observed in GD. Such findings would provide a new perspective on the neurobiology of GD, beyond isolated regional abnormalities, by highlighting network-level dysfunction. In turn, identifying a coherence-based signature of GD could pave the way for developing EEG biomarkers for clinical monitoring or for targeting neuromodulation therapies to normalize functional connectivity in this population. Interhemispheric coherence, defined as the synchrony between homologous electrode pairs across the hemispheres, is of particular interest because it reflects the integrity of transcallosal communication. Homologous sites are thought to provide the most direct scalp-level index of hemispheric integration (Bloom and Hynd, 2005). Prior studies have demonstrated that abnormalities in interhemispheric coherence occur in several developmental and psychiatric conditions, including ADHD and schizophrenia (Gasser et al., 1982; Clarke et al., 2005), suggesting that disrupted callosal connectivity may have clinical relevance. By focusing on these measures, our study provides a complementary perspective to prior work on frontal asymmetry and fronto–temporal connectivity, emphasizing hemispheric balance and integration as potential mechanisms in GD.

## 2 Materials and methods

### 2.1 Study design

This retrospective, cross-sectional, case–control study compared resting-state qEEG coherence between individuals diagnosed with GD and healthy controls (HCs). Data were obtained from previously recorded EEG assessments conducted under standardized laboratory conditions. The primary objective was to identify group differences in interhemispheric coherence across canonical frequency bands (delta, theta, alpha, beta).

### 2.2 Participants

The study included a total of 74 adults, consisting of 29 individuals with GD and 45 HCs. All data were obtained retrospectively from individuals who presented to NP Istanbul Neuro Psychiatry Hospital, the clinical branch of Üsküdar University, for clinical evaluation. The GD group comprised patients diagnosed by psychiatrists according to DSM-5 criteria for GD during routine outpatient addiction clinic assessments. The HCs group consisted of individuals who attended the hospital for general health evaluations and had no history of psychiatric or neurological disorders. Eligible participants in both groups were between 18 and 65 years of age, right-handed, native Turkish speakers, and had normal or corrected-to-normal vision. Exclusion criteria for both groups included any history of neurological disease, traumatic brain injury, or seizure disorder; substance dependence other than nicotine within the past year; and current use of medications known to significantly affect EEG activity (e.g., benzodiazepines). In addition, individuals with a clinical diagnosis of nicotine dependence were excluded from both groups, although occasional non-dependent nicotine use was not considered an exclusion criterion. For the HCs group, additional exclusion criteria were a history of psychiatric disorders or a family history of pathological gambling. The study was approved by the Üsküdar University (including NP Hospital) institutional ethics committee, and all procedures were conducted in accordance with the Declaration of Helsinki. For retrospective data, consent for use of anonymized clinical information in research was obtained at the time of evaluation.

### 2.3 Measures

#### 2.3.1 South oaks gambling screen – Turkish version (SOGS)

The South Oaks Gambling Screen (SOGS) was originally developed by Lesieur and Blume (1987) as a 20-item self-report measure to identify pathological gambling. The Turkish adaptation was carried out by Duvarci and Varan (2001) in two separate validation studies. During adaptation, three original items that did not discriminate pathological gamblers from controls in the Turkish context were removed and replaced with two culturally relevant items (e.g., borrowing from friends or converting gold/jewelry to cash). The final Turkish form consists of 19 scored items, with a cut-off score of 8 points yielding optimal sensitivity and specificity (both = 90%) for identifying probable pathological gamblers according to DSM-IV criteria. Internal consistency was high (Cronbach's α = 0.88) and test–retest reliability over 1 month was excellent (*r* = 0.95). Scores range from 0 to 19, with higher scores indicating greater gambling-related problems; individuals scoring 8 or more are classified as probable pathological gamblers.

#### 2.3.2 Beck anxiety inventory (BAI)

The Beck Anxiety Inventory (BAI) was developed by Beck et al. (1993) to assess the severity of clinical anxiety. The Turkish adaptation was performed by Ulusoy et al. (1998), following translation and back-translation procedures. The 21 items are rated on a 4-point scale (0–3), with total scores ranging from 0 to 63, focusing primarily on the physiological and somatic symptoms of anxiety. Exploratory factor analysis of the Turkish version yielded two factors, and the instrument demonstrated excellent internal consistency (α = 0.93) and acceptable test–retest reliability (*r* = 0.75). Convergent validity was supported through moderate correlations with the Beck Depression Inventory and other established anxiety measures.

#### 2.3.3 Beck depression inventory-II (BDI-II)

The Beck Depression Inventory-II (BDI-II) is a 21-item self-report measure of depressive symptoms based on DSM-IV criteria, rated on a 0–3 scale with total scores ranging from 0 to 63. The Turkish adaptation (BDI-II-TR) was validated by Kapci et al. (2008) in clinical (*n* = 176) and nonclinical (*n* = 362) adult samples, showing high internal consistency (α = 0.90 nonclinical; α = 0.89 clinical) and excellent 2-week test–retest reliability (*r* = 0.94). Convergent validity was supported by strong correlations with the original BDI (*r* = 0.82) and BSI-Depression (*r* = 0.67). Factor analyses in the clinical sample confirmed a two-factor structure (“somatic/affective” and “cognitive”), explaining 40.7% of the variance. ROC analysis established cut-off scores of 0–12 (minimal), 13–18 (mild), 19–28 (moderate), and 29–63 (severe), consistent with the original classification.

#### 2.3.4 qEEG coherence

Quantitative EEG coherence was computed to assess functional connectivity between cortical regions in both GD and HCs groups. Mathematically, coherence is derived from the cross-spectrum of two EEG signals and yields a value between 0 and 1, where 1 indicates a perfect linear phase relationship at a given frequency and 0 indicates no consistent relationship (Essl and Rappelsberger, 1998). In the present study, coherence values were expressed in percentage form (0–100) as output by the Neuroguide software, with higher percentages reflecting stronger phase synchrony between electrode pairs. For each participant, a continuous, artifact-free 180-s (3-min) resting-state epoch was selected from the longer recording and used for connectivity analysis (Miljevic et al., 2022). Coherence values were derived via Fast Fourier Transform (FFT)–based cross-spectral analysis for delta (1–4 Hz), theta (4–8 Hz), alpha (8–12 Hz), and beta (12–25 Hz) bands. Gamma (>30 Hz) was not analyzed due to its susceptibility to muscle and ocular artifacts in scalp EEG recordings. Interhemispheric coherence was calculated between homologous electrode pairs (Fp1–Fp2, F3–F4, F7–F8, C3–C4, P3–P4, T3–T4, T5–T6, O1–O2).

Coherence was selected as the primary connectivity measure because it simultaneously captures both amplitude and phase synchrony and is widely applied in clinical EEG research, particularly in psychiatric and addiction studies. While scalp coherence is inevitably influenced by volume conduction, spurious correlations are most problematic for short-range intrahemispheric pairs where overlapping fields are greatest. Interhemispheric coherence between homologous left–right sites, although not entirely immune (e.g., C3–C4 is closer than some long-range intrahemispheric pairs such as F3–O1), provides a more interpretable index of large-scale functional connectivity and is thought to reflect transcallosal communication (Essl and Rappelsberger, 1998; Nunez and Srinivasan, 2006; Miljevic et al., 2022). On this basis, we prioritized interhemispheric coherence as our primary metric, while noting that future work employing source-space methods or current-source density approaches could further minimize volume conduction effects.

### 2.4 qEEG recording and analysis procedure

All EEG recordings were conducted in a quiet, dimly lit, electrically shielded, and sound-attenuated room. Participants were seated comfortably and instructed to remain relaxed with their eyes closed, minimizing blinking and other movements to reduce ocular and muscle artifacts. Resting-state EEG data were acquired using Neuroguide software (version 2.5) under eyes-closed conditions for 9–20 min. Nineteen scalp electrodes were positioned according to the international 10–20 system, with linked mastoid electrodes (A1 and A2) serving as references during acquisition. Active electrodes embedded within a cap were used, and impedance was maintained below 5 kΩ. EEG signals were sampled at 250 Hz, bandpass filtered between 0.15–70 Hz, and notch filtered at 50 Hz to remove power line interference. Artifacts were removed offline through manual inspection to exclude blink and eye movement contamination. From these preprocessed data, a continuous, artifact-free, 3-min epoch was selected for each participant and entered into coherence analysis. Coherence values were then aggregated by cerebral lobes—midline interhemispheric (Fz, Cz, Pz), parietal (P3, P4), occipital (O1, O2), frontal (F3, F4, F7, F8), and temporal (T3, T4, T5, T6)—to enhance interpretability while reducing dimensionality.

### 2.5 Statistical analysis

Prior to between-group comparisons, Levene's F-test was used to assess homogeneity of variances for age, and the GD and HC groups were matched on age accordingly. Descriptive data are presented as mean ± standard deviation (M ± SD). Group differences in coherence values were analyzed using independent-samples *t*-tests at each interhemispheric electrode pair. Effect sizes for these comparisons were calculated using Cohen's *d*, interpreted as small (0.20), medium (0.50), and large (0.80) effects. Given the modest sample size, we did not employ multivariate approaches such as ANOVA, as this would result in very few observations per electrode pair and unstable estimates. Similarly, we did not apply formal multiple-comparison corrections across all tests, since our design involved a series of predefined, independent two-group comparisons rather than exploratory all-to-all contrasts. To mitigate concerns about false positives, we emphasized consistent regional patterns across frequency bands and reported effect sizes alongside *p*-*values* to provide a balanced interpretation of statistical outcomes. Associations between coherence measures and clinical variables were examined using Pearson correlation coefficients. Prior to conducting these analyses, assumptions of Pearson correlation were checked: the variables used (e.g., SOGS, BDI-II, BAI scores, and EEG coherence values) were continuous, approximately normally distributed, and linearly related. For robustness, we also verified that the overall pattern of results was consistent when using Spearman's rho. Partial correlations were further conducted to control for potential age effects, ensuring that observed relationships were not confounded by group age differences (Tarlaci and Hidimoglu, 2025). All statistical tests were two-tailed with a significance threshold set at *p* < 0.05, and 95% confidence intervals (CIs) were reported where appropriate. Analyses were performed using SPSS Statistics (version 27) and JASP (version 0.9).

## 3 Results

### 3.1 Demographics and clinical characteristics

All participants were male. Groups were age-matched after verifying homogeneity of variances using Levene's F-test. The GD and HCs groups were similar in basic demographics. The mean age was 32.86 ± 10.11 years for the GD group and 35.13 ± 8.70 years for the HCs group, with no significant difference between them [*t*_(72)_ = −1.03, *p* = 0.305]. The GD group had a mean disorder duration of 7.03 ± 4.19 years (range: 1–18 years). In terms of comorbidity, 13.8% (*n* = 4) of GD participants had at least one co-occurring psychiatric disorder; 10.3% (*n* = 3) had ADHD and 3.4% (*n* = 1) had Obsessive-Compulsive Disorder (OCD). No participants in the HCs group had any psychiatric diagnoses (by study design). Most GD participants (=79%) had attained at least a high school education, and there were no significant group differences in years of education (qualitatively assessed). Detailed demographic and clinical characteristics are presented in Table 1.

**Table 1:** Demographic and clinical characteristics of GD and HCs participants.

**Characteristic**	**GD (*n* = 29)**	**HCs (*n* = 45)**	**Test statistic**	***p***
Age (years), M ± SD	32.86 ± 10.11	35.13 ± 8.70	*t*_(72)_ = −1.03	0.30
Disorder duration (years), M ± SD^a^	7.03 ± 4.19 (range 1–18)	—	—	—
Any psychiatric comorbidity, %^a^	13.8	0	—	—

GD, Gambling Disorder; HCs, Healthy Controls. t-test is two-tailed.

^a^Disorder duration and comorbidity are applicable only to the GD group.

### 3.2 EEG coherence group comparisons

**Figure 1:**
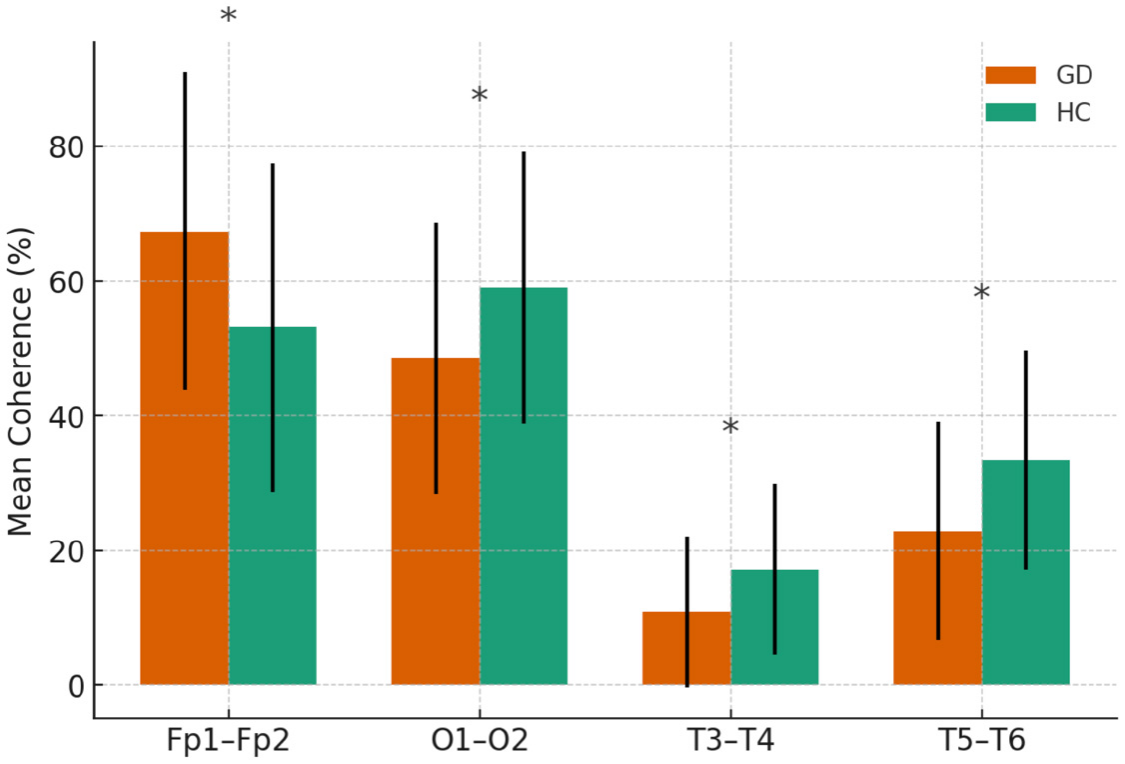
Delta-band coherence in GD and HCs groups.

**Figure 2:**
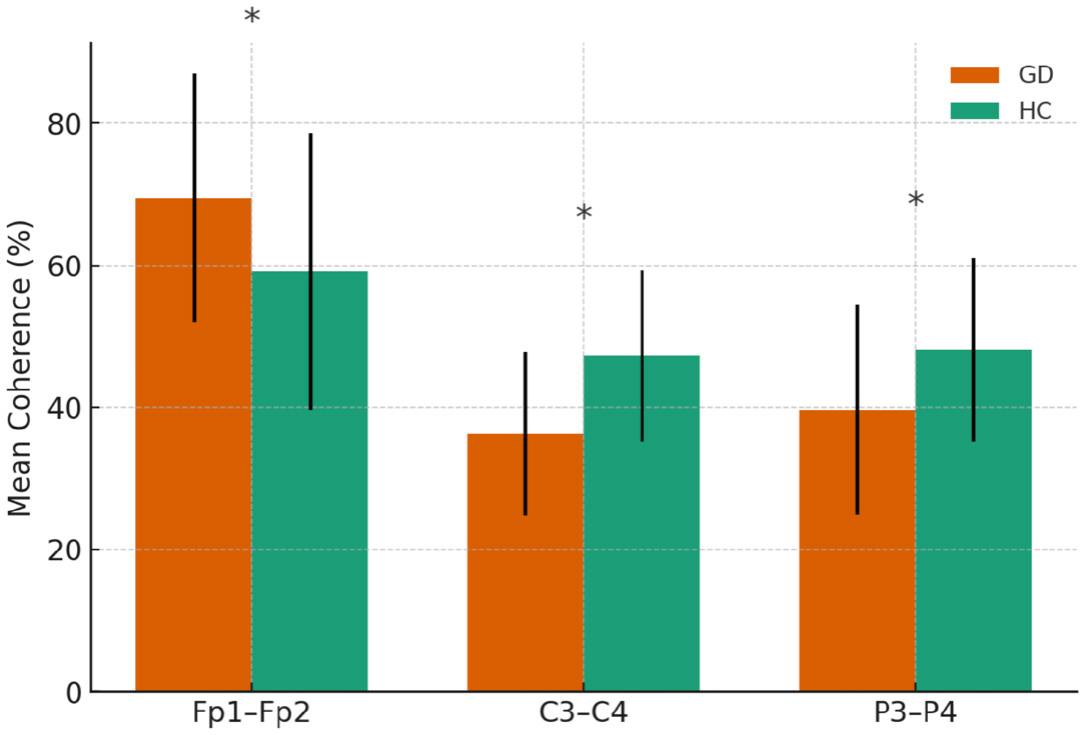
Beta-band coherence in GD and HCs groups. Asterisks indicate significant group differences (*p* < 0.05)

Group differences in resting-state qEEG coherence were analyzed for each frequency band using independent-samples *t*-tests (Tables 2–5). Figure 1 through Figure 2 illustrates the significant coherence differences between GD and HCs participants in each band.

**Table 2:** Independent-samples *t*-tests—delta band coherence (GD vs. HCs).

**Electrode pair**	**GD M ±SD**	**HC M ±SD**	***t*(df)**	***d***	***p***
Fp1–Fp2	67.41 ± 23.58	53.13 ± 24.39	2.49 (72)	0.59	0.015
C3–C4	52.66 ± 22.87	60.69 ± 19.31	−1.63 (72)	−0.39	0.109
O1–O2	48.63 ± 20.13	59.04 ± 20.18	−2.17 (72)	−0.52	0.034
T3–T4	10.90 ± 11.23	17.24 ± 12.65	−2.20 (72)	−0.52	0.031
F3–F4	58.79 ± 19.23	66.29 ± 14.93	−1.78 (49.26)^a^	−0.45	0.081
P3–P4	52.68 ± 23.05	62.11 ± 20.81	−1.82 (72)	−0.43	0.072
F7–F8	22.78 ± 17.45	27.06 ± 17.48	−1.03 (72)	−0.25	0.307
T5–T6	22.96 ± 16.21	33.45 ± 16.32	−2.71 (72)	−0.64	0.009

GD, Gambling Disorder; HCs, Healthy Controls; M, Mean; SD, Standard Deviation.

Positive t indicates GD > HC. d = Cohen's d.

^a^Equal variances not assumed (Welch's t reported).

**Table 3:** Independent-samples *t*-tests—theta band coherence (GD vs. HCs).

**Electrode pair**	**GD M ±SD**	**HCs M ±SD**	***t*(df)**	***d***	***p***
Fp1–Fp2	73.2 ± 18.1	64.0 ± 19.7	2.03 (72)	0.48	0.046
C3–C4	53.8 ± 16.1	58.7 ± 17.4	−1.22 (72)	−0.29	0.225
O1–O2	54.9 ± 16.8	55.0 ± 17.7	−0.04 (72)	−0.01	0.971
T3–T4	5.57 ± 6.68	9.26 ± 8.60	−1.96 (72)	−0.47	0.054
F3–F4	63.6 ± 13.7	68.6 ± 11.4	−1.69 (72)	−0.40	0.095
P3–P4	51.4 ± 17.6	59.9 ± 17.7	−2.03 (72)	−0.48	0.046
F7–F8	23.6 ± 16.5	24.7 ± 14.0	−0.31 (72)	−0.07	0.756
T5–T6	15.3 ± 11.7	20.1 ± 12.1	−1.69 (72)	−0.40	0.096

**Table 4:** Independent-samples *t*-tests—alpha band coherence (GD vs. HC).

**Electrode pair**	**GD M ±SD**	**HCs M ±SD**	***t*(df)**	***d***	***p***
Fp1–Fp2	85.36 ± 13.77	83.18 ± 13.01	0.69 (72)	0.16	0.494
C3–C4	49.60 ± 16.81	63.18 ± 16.60	−3.42 (72)	−0.81	0.001
O1–O2	61.57 ± 17.09	60.20 ± 17.71	0.33 (72)	0.08	0.742
T3–T4	5.48 ± 5.56	12.51 ± 13.88	−3.04 (62.57)^a^	−0.62	0.003
F3–F4	76.18 ± 13.63	80.88 ± 9.68	−1.62 (46.07)^a^	−0.41	0.113
P3–P4	49.59 ± 18.35	60.69 ± 15.17	−2.83 (72)	−0.67	0.006
F7–F8	43.14 ± 23.02	44.93 ± 18.92	−0.37 (72)	−0.09	0.716
T5–T6	20.76 ± 15.93	24.12 ± 15.57	−0.90 (72)	−0.21	0.371

GD, Gambling Disorder; HCs, Healthy Control; M, Mean; SD, Standard Deviation.

d = Cohen's d. ^a^Equal variances not assumed (Welch's t reported).

**Table 5:** Independent-samples *t*-tests—beta band coherence (GD vs. HCs).

**Electrode pair**	**GD M ±SD**	**HC M ±SD**	***t*(df)**	***d***	***p***
Fp1–Fp2	69.4 ± 17.5	59.1 ± 19.5	2.32 (72)	0.55	0.023
C3–C4	36.2 ± 11.4	47.2 ± 12.1	−3.89 (72)	−0.93	0.001
O1–O2	51.8 ± 13.0	49.4 ± 11.9	0.82 (72)	0.19	0.418
T3–T4	3.39 ± 5.94	3.62 ± 6.96	−0.15 (72)	−0.04	0.882
F3–F4	55.5 ± 11.9	59.4 ± 12.9	−1.28 (72)	−0.31	0.203
P3–P4	39.7 ± 14.8	48.1 ± 13.0	−2.57 (72)	−0.61	0.012
F7–F8	17.3 ± 13.8	21.0 ± 11.1	−1.26 (72)	−0.30	0.212
T5–T6	9.60 ± 7.82	8.65 ± 7.56	0.52 (72)	0.12	0.607

#### 3.2.1 Interhemispheric delta coherences

In the delta band, the GD group showed significantly higher interhemispheric coherence than HCs at Fp1–Fp2 (67.4 ± 23.6 vs. 53.1 ± 24.4, *p* = 0.015). In contrast, GD participants had lower coherence at O1–O2 (48.6 ± 20.1 vs. 59.0 ± 20.2, *p* = 0.034), T3–T4 (10.9 ± 11.2 vs. 17.2 ± 12.7, *p* = 0.031), and T5–T6 (23.0 ± 16.2 vs. 33.4 ± 16.3, *p* = 0.009). Other electrode pairs showed no significant group differences, although F3–F4 and P3–P4 displayed non-significant trends toward lower coherence in the GD group (*p* = 0.06–0.08).

In Figure 1, mean delta-band coherence (± SE) at four interhemispheric electrode pairs (Fp1–Fp2, O1–O2, T3–T4, T5–T6) for the GD and HCs groups. Asterisks indicate significant group differences (*p* < 0.05).

#### 3.2.2 Interhemispheric theta coherences

Frontopolar coherence was higher in the GD group at Fp1–Fp2 (73.2 ± 18.1 vs. 64.0 ± 19.7, *p* = 0.046), while parietal coherence at P3–P4 was lower (51.4 ± 17.6 vs. 59.9 ± 17.7, *p* = 0.046). A trend toward lower temporal coherence at T3–T4 was observed in the GD group (5.57 ± 6.68 vs. 9.26 ± 8.60, *p* = 0.054). No other electrode pairs showed significant group differences.

**Figure 3:**
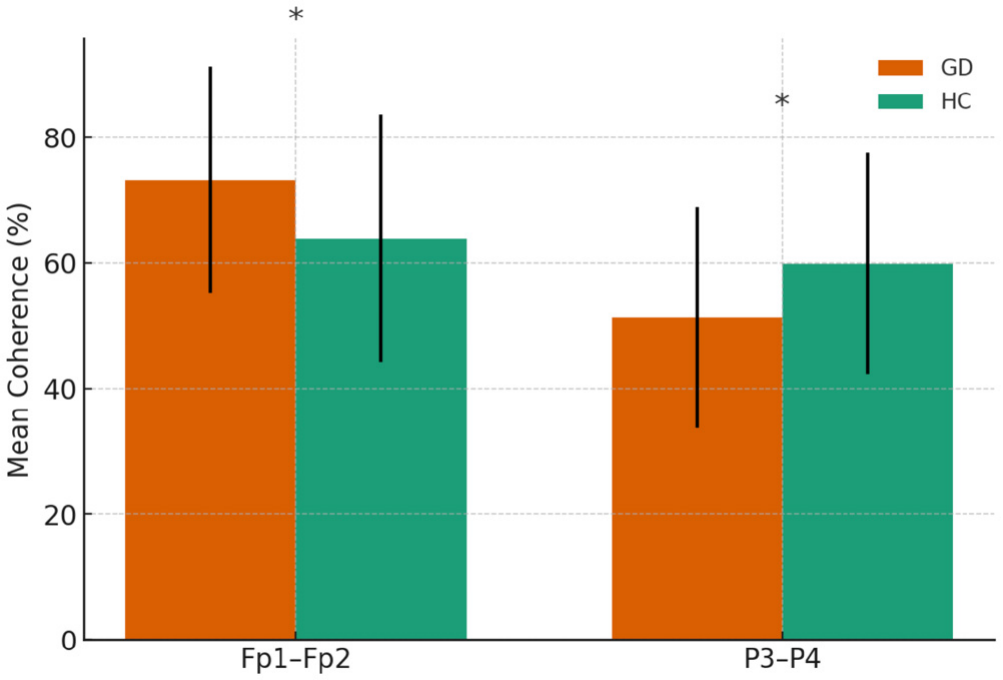
Theta-band coherence in GD and HCs groups. Asterisks indicate significant group differences (*p* < 0.05)

In Figure 3, mean theta-band coherence (± SE) at significant electrode pairs for GD vs. HCs (Fp1–Fp2 and P3–P4). No significant group difference was found at T3–T4 (shown for reference).

#### 3.2.3 Interhemispheric alpha coherences

All significant differences reflected lower coherence in the GD group. Coherence was reduced at C3–C4 (49.6 ± 16.8 vs. 63.2 ± 16.6, *p* = 0.001), T3–T4 (5.5 ± 5.6 vs. 12.5 ± 13.9, *p* = 0.003), and P3–P4 (49.6 ± 18.4 vs. 60.7 ± 15.2, *p* = 0.006). No other electrode pairs showed significant differences.

**Figure 4:**
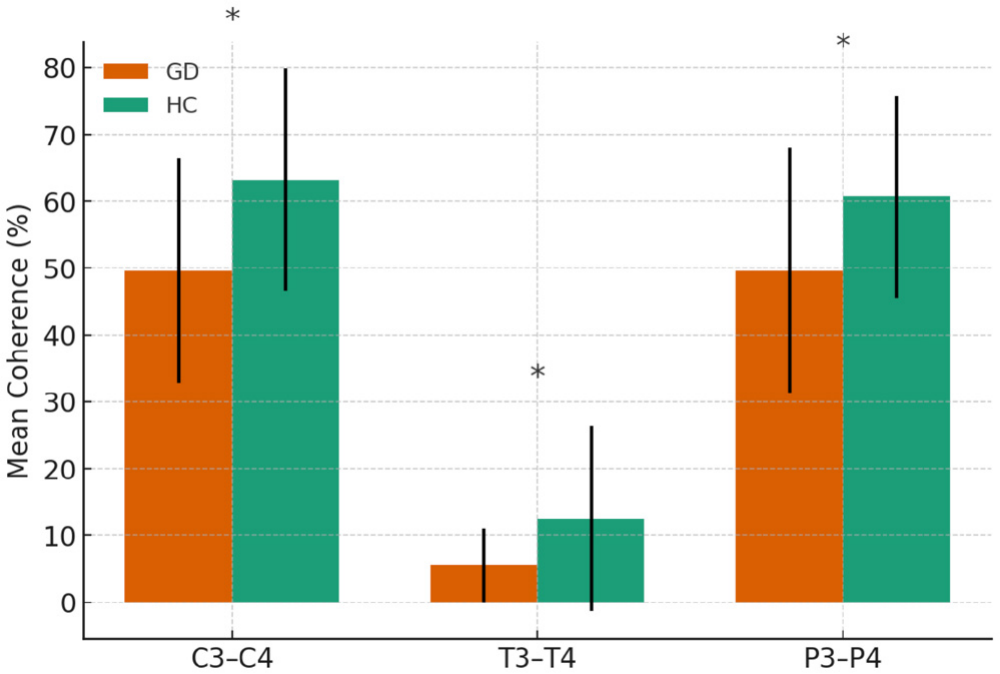
Alpha-band coherence in GD and HCs groups. Asterisks indicate significant group differences (*p* < 0.05)

In Figure 4, mean alpha-band coherence (± SE) at electrode pairs with significant group differences (C3–C4, T3–T4, P3–P4). GD group consistently showed lower alpha coherence at these pairs compared to HCs.

#### 3.2.4 Interhemispheric beta coherences

The GD group showed higher coherence at Fp1–Fp2 (69.4 ± 17.5 vs. 59.1 ± 19.4, *p* = 0.023) but lower coherence at C3–C4 (36.2 ± 11.4 vs. 47.2 ± 12.1, *p* = 0.001) and P3–P4 (39.7 ± 14.8 vs. 48.1 ± 13.0, *p* = 0.012). No other electrode pairs showed significant group differences.

In Figure 2, mean beta-band coherence (± SE) at electrode pairs with significant group differences (Fp1–Fp2, C3–C4, P3–P4). The GD group exhibited higher frontopolar beta coherence but lower central and parietal beta coherence relative to controls.

**Figure 5:**
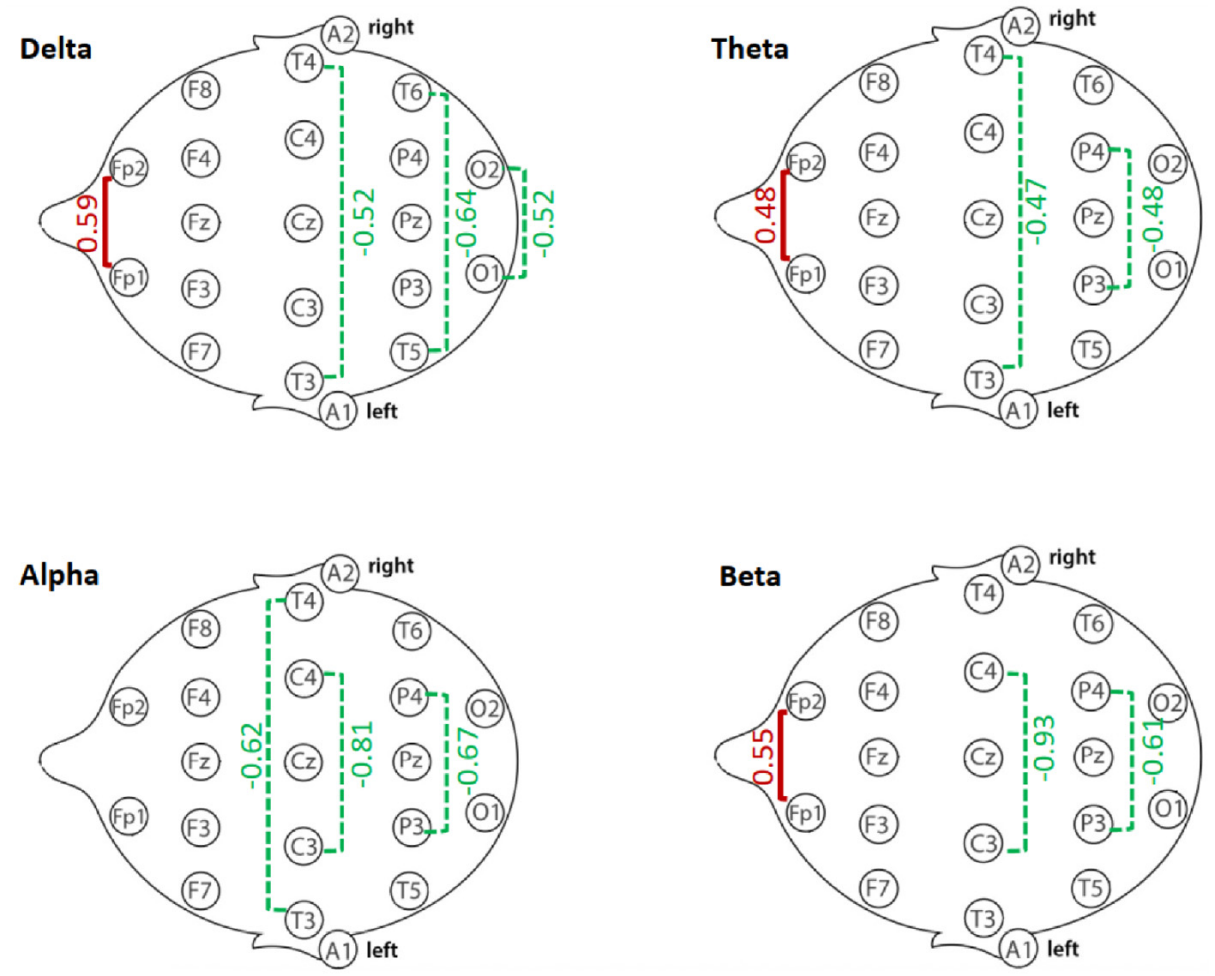
Summary of interhemispheric coherence differences (Cohen's *d*) between GD and Healthy Controls (HCs) groups across frequency bands. Positive effect sizes (red) indicate higher coherence in GD compared to HCs; negative effect sizes (green) indicate lower coherence in GD compared to HCs. Values reflect Cohen's d from independent-samples *t*-tests for each electrode pair.

Figure 5 summarizes the magnitude and direction of interhemispheric coherence differences between GD and HCs participants across delta, theta, alpha, and beta bands. Effect sizes revealed a mixed pattern: GD participants demonstrated increased frontopolar coherence in delta, theta, and beta bands, but reduced coherence in central, parietal, temporal, and occipital regions across several bands. The visualization highlights the regional specificity of these alterations, with the largest reductions observed in central alpha and beta coherence.

### 3.3 Coherences and relationship with gambling severity (SOGS)

Pearson correlation analyses revealed no significant associations between resting-state coherence and gambling severity as measured by the SOGS in the GD group. Across all frequency bands and electrode pairs, none of the coherence measures showed a correlation with SOGS scores that reached significance (all *p* > 0.05; see Table 6). Correlation coefficients were generally small in magnitude (|*r*| < 0.30). For example, SOGS scores were not significantly related to frontal delta coherence (e.g., *r* = 0.20, *p* = 0.29 for Fp1–Fp2) or parietal alpha coherence (*r* = 0.10, *p* = 0.61 for P3–P4). The largest observed correlation was a positive trend between SOGS and posterior-temporal alpha coherence (T5–T6: *r* = 0.31, *p* = 0.11), but this did not reach significance. Thus, higher gambling severity (SOGS score) was not associated with any systematic increase or decrease in interhemispheric coherence in this sample.

**Table 6:** Pearson correlations of coherence with SOGS scores (GD Group).

**Electrode pair**	**Delta r (p)**	**Theta r (p)**	**Alpha r (p)**	**Beta r (p)**
Fp1–Fp2	0.20 (0.293)	0.24 (0.217)	0.29 (0.124)	0.10 (0.597)
C3–C4	0.08 (0.700)	0.23 (0.237)	0.15 (0.445)	0.00 (0.993)
O1–O2	0.06 (0.764)	−0.01 (0.962)	−0.12 (0.549)	−0.15 (0.442)
T3–T4	0.01 (0.954)	−0.02 (0.906)	0.06 (0.745)	−0.11 (0.584)
F3–F4	0.07 (0.733)	0.16 (0.396)	0.14 (0.473)	0.02 (0.935)
P3–P4	0.05 (0.795)	0.10 (0.613)	0.03 (0.877)	0.01 (0.951)
F7–F8	−0.00 (0.992)	0.03 (0.873)	0.15 (0.436)	−0.18 (0.359)
T5–T6	0.01 (0.954)	0.09 (0.630)	0.16 (0.412)	−0.14 (0.482)

GD, Gambling Disorder; Delta, Delta band; Theta, Theta band; Alpha, Alpha band; Beta, Beta band. Pearson's r values are shown with two-tailed p-values in parentheses.

### 3.4 Coherences and relationship with disorder duration

**Figure 6:**
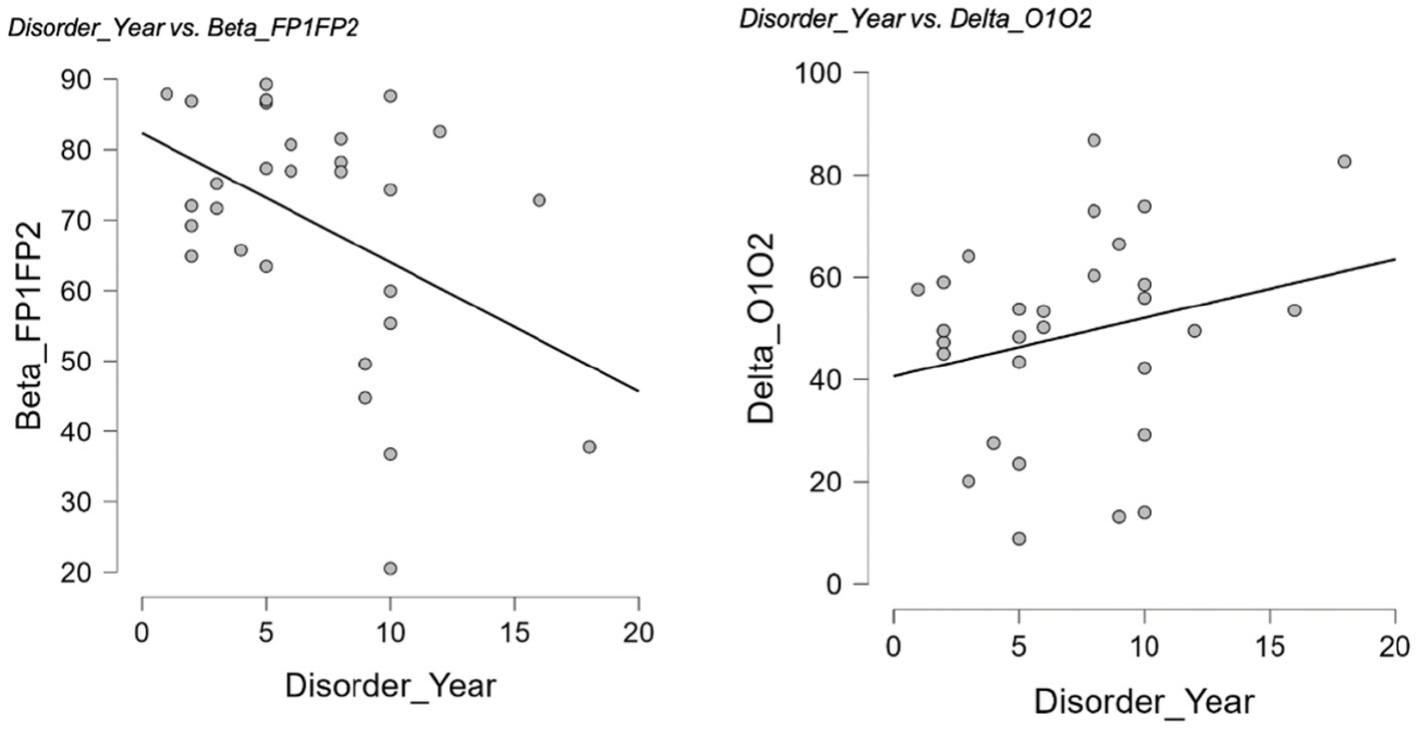
Partial correlations between years of GD and interhemispheric coherence. Scatterplots illustrate significant partial correlations between years of D and interhemispheric coherence after controlling for age. Left: Longer disorder duration was associated with lower beta coherence at Fp1–Fp2 (*r* = −0.39, *p* = 0.041). Right: Longer disorder duration was associated with higher delta coherence at O1–O2 (*r* = 0.40, *p* = 0.034).

Within the GD group, resting-state coherence showed several significant partial correlations with disorder duration (years of GD) when controlling for age (Table 7). Longer duration of gambling disorder was associated with lower beta-band coherences in frontal and central regions. In particular, disorder length correlated negatively with interhemispheric beta coherence at F3–F4 (*r* = −0.51, *p* = 0.005)—indicating that patients with more years of GD had significantly reduced frontal (midline) beta connectivity. Similarly, beta coherence was negatively related to disorder duration at Fp1–Fp2 (*r* = −0.39, *p* = 0.041) and F7–F8 (*r* = −0.39, *p* = 0.041), reflecting lower frontopolar and lateral-frontal beta coherence in longer-term GD. In contrast, a positive partial correlation emerged in the delta band: longer GD duration was modestly associated with higher delta coherence between occipital regions O1–O2 (*r* = 0.40, *p* = 0.034). These relationships remained significant after accounting for participants' age. Scatterplots illustrating two of these partial correlations are presented in Figure 6. No other coherence measures showed significant partial correlations with disorder duration after age control (all other r values *p* > 0.05).

**Table 7:** Significant partial correlations of coherence with disorder duration (Age-Controlled, GD Group).

**Coherence measure (Band)**	**Partial *r***	***p***
Beta F3–F4 (Frontal)	−0.51	0.005
Beta Fp1–Fp2 (Frontopolar)	−0.39	0.041
Beta F7–F8 (Frontal Lateral)	−0.39	0.041
Delta O1–O2 (Occipital)	0.40	0.034

Pearson partial correlations (two-tailed) controlling for age, among GD participants. Only measures with *p* < 0.05 are shown. Positive r indicates higher coherence with longer disorder duration; negative r indicates lower coherence with longer duration.

Interestingly, patients with longer GD duration in some cases showed a more “healthy control–like” coherence profile. This may reflect compensatory neural mechanisms that stabilize interhemispheric connectivity over time, or selection effects, whereby individuals with less stable neural dynamics are underrepresented among long-duration cases. Methodological factors such as subtle variability in adherence or recording conditions cannot be excluded. Future longitudinal studies are needed to determine whether this pattern reflects genuine neural adaptation or sample-related influences.

Within the GD group, partial correlations controlling for age revealed that longer disorder duration was associated with lower beta coherence at Fp1–Fp2 (*r* = −0.39, *p* = 0.041) and higher delta coherence at O1–O2 (*r* = 0.40, *p* = 0.034; Figure 6).

Within the GD group, as shown in Table 7, partial correlations controlling for age indicated that longer disorder duration was associated with lower beta coherence at F3–F4 (*r* = −0.51, *p* = 0.005), Fp1–Fp2 (*r* = −0.39, *p* = 0.041), and F7–F8 (*r* = −0.39, *p* = 0.041), and with higher delta coherence at O1–O2 (*r* = 0.40, *p* = 0.034).

## 4 Discussion

Consistent with our a priori hypothesis, GD was associated with a dual pattern of altered interhemispheric functional connectivity, showing both hypercoherence and hypocoherence across distinct cortical regions and frequency bands. As predicted, slow-wave coherence (delta, theta) and beta-band connectivity were particularly affected, with increases observed in frontopolar regions and decreases in central, parietal, and temporal areas. Notably, we observed robust hypercoherence in the frontal pole, alongside hypocoherence across temporal, central, and parietal regions when compared to healthy controls. At the frontal pole, GD participants showed significantly elevated coherence between Fp1–Fp2 (left and right frontopolar EEG leads) in the delta, theta, and beta bands. These scalp sites are positioned over anterior prefrontal regions and are likely influenced by underlying prefrontal and orbitofrontal generators, although precise source localization cannot be inferred from scalp EEG alone (Nunez and Srinivasan, 2006; Koessler et al., 2009). The hyper-synchronization observed at Fp1–Fp2 may therefore reflect increased integration within frontal regulatory circuits involved in decision making, reward evaluation, and impulse control. Such excessive coupling could indicate reduced network flexibility, potentially contributing to the impaired self-regulation that characterizes GD. In functional terms, this hypercoherence could indicate that the two hemispheric poles of the prefrontal cortex are over-integrated during rest, possibly contributing to the impaired decision-making and loss of impulse control characterizing GD. Excessive coupling in orbitofrontal networks might limit the flexibility of frontal systems to adapt or segregate during cognitive processing, potentially manifesting as the compulsive, perseverative decision patterns seen in problem gambling. This interpretation is consistent with prior studies suggesting that abnormally high coherence in frontal regions can be maladaptive. For instance, Lee et al. (2017) found that among individuals with gaming disorder, those with lower resilience (a protective psychological factor) tended to have higher resting alpha coherence, whereas higher-resilience individuals showed more normalized (lower) coherence. Such an inverse relationship between resilience and coherence supports the notion that increased interhemispheric coupling (particularly in frontal networks) may signify a less favorable neural profile in addiction, perhaps reflecting rigid network dynamics or a lack of efficient hemispheric specialization. Our finding of frontopolar hypercoherence in GD aligns with this view, suggesting it could be a neural marker of the impaired self-regulatory control that predisposes to addictive behavior. In line with this, Park et al. (2017) compared IGD, Alcohol Use Disorder, and HCs, and reported that IGD patients exhibited significantly increased intrahemispheric gamma coherence, particularly in fronto-central regions, and that this fast-frequency coherence predicted addiction severity. These findings highlight that hypercoherence in frontal-related networks may represent a broader marker of behavioral addictions, though the specific frequency bands involved differ across condition. Our results of increased frontopolar beta coherence in GD parallel findings by Park et al. (2018), who reported increased interhemispheric beta and gamma coherence in IGD. Both studies suggest that heightened fast-frequency synchrony may be a common feature of behavioral addictions, although GD additionally showed decreased beta coherence in posterior regions. This may indicate that while frontal hypercoherence is a shared marker of addiction vulnerability, posterior hypocoherence may be more specific to GD, potentially reflecting impairments in attentional and sensorimotor integration.

In contrast to the frontal hypercoherence, interhemispheric coherence was reduced in GD across several other cortical regions, consistent with our expectation of posterior and association-area hypoconnectivity. We found significant hypocoherence between temporal, central, and parietal electrode pairs in the GD group. For example, coherence was lower between bilateral temporal regions (as indexed by connections such as T3–T4, roughly over mid-temporal cortex, and T5–T6 over temporo-occipital cortex). These electrodes correspond to associative temporal areas (Brodmann areas 21/22/42 for mid-temporal; BA19/37/38 for temporo-occipital regions) involved in auditory processing, language and semantic memory, as well as visual–limbic integration. Diminished coupling between the left and right temporal lobes in GD suggests a functional disconnection in circuits that integrate sensory cues and mnemonic or emotional context. In practical terms, such hypocoherence might impair how individuals with GD process environmental stimuli and contextual information during decision-making (Langham et al., 2017). For instance, reduced synchronization in temporal association areas could contribute to aberrant cue-reactivity—gamblers might not effectively integrate contextual cues (e.g., odds, consequences) with learned emotional or memory inputs, potentially facilitating biased attention toward gambling-related cues or diminished evaluation of losses.

We also observed reduced coherence between central (C3–C4) and parietal (P3–P4) electrode pairs in the GD group, consistent with our hypothesis of diminished beta-band connectivity in sensorimotor and attention networks. C3–C4 overlie the primary sensorimotor cortices (approximate BA 3/1/4), while P3–P4 cover lateral parietal regions (including BA 7, 19, 40, spanning the superior parietal cortex, visual association cortex, and inferior parietal lobule). Hypocoherence in the central region implies that the normally coordinated activity between left and right sensorimotor cortices is weakened in GD. Although the motor cortex is not classically associated with addiction pathology (Kalivas and Volkow, 2005), this finding may reflect a more global neural dysregulation or altered arousal in GD, and it could relate to subtle deficits in interhemispheric coordination of actions. There is some evidence that motor cortical connectivity is involved in inhibitory control (for example, successful response inhibition requires bilateral motor cortex communication); thus, reduced C3–C4 coherence might hint at weaker interhemispheric integration underlying motor aspects of impulse control (Rae et al., 2015). Similarly, the decreased parietal coherence observed (P3–P4) points to a disruption in bilateral parietal lobe networks that govern visuospatial attention and the integration of sensory information (Abramov et al., 2017). The parietal cortices are critical for attentional control and shifting focus (Shomstein, 2012); thus, their disconnection in GD could contribute to the attentional inflexibility or neglect of negative consequences often seen in problem gamblers (Ciccarelli et al., 2016). In sum, the pattern of regional hypocoherence—spanning temporal association areas, somatosensory cortex, and parietal attention networks—suggests that GD is marked by selective under-connectivity in neural systems responsible for integrating sensory, spatial, and executive information. This may translate to difficulties in holistically appraising gambling situations (e.g., failing to integrate odds and past losses with ongoing behavior) and a breakdown in the smooth coordination between intention (frontal) and action (sensorimotor) that is necessary for self-control.

Our results can be interpreted in light of prior EEG coherence studies in addiction, which reveal both consistencies and divergences, particularly regarding the frontal hypercoherence component anticipated in our hypothesis. Hypercoherence in frontal regions has been noted in some studies of behavioral addiction, though often in different frequency bands. Lee et al. (2017), for example, examined individuals with IGD (a condition behaviorally akin to gambling addiction) and reported increased coherence in higher-frequency bands in those patients. They found that intrahemispheric coherence was significantly elevated in the gaming disorder group (relative to both alcohol-dependent and healthy comparison groups), leading them to propose increased fast-frequency synchrony as a potential trait marker of that addiction. Although that study did not detect interhemispheric coherence changes in the gaming addicts, the presence of heightened connectivity in frontal-related networks (right fronto-central coherence, specifically) parallels our observation of enhanced frontopolar coherence in GD, suggesting that hyper-connectivity in frontal networks may be a common neurophysiological feature across behavioral addictions. It is worth noting, however, that the frequencies differ (higher-frequency activity in gaming disorder vs. delta/theta/beta in our GD sample), which could reflect modality-specific differences or methodological factors. Interestingly, Lee et al. (2017) also reported that the elevated coherence in gamers showed a positive correlation with addiction severity (Internet Addiction Test scores), implying that stronger neural coupling was associated with more severe addictive behavior. In our gambling study, we examined correlations between coherence and clinical indices. Gambling severity as measured by the SOGS did not show significant associations with coherence, but disorder duration was significantly related to several coherence measures (e.g., lower frontal beta and higher occipital delta coherence with longer illness duration). We did not test depressive or anxiety symptoms in relation to coherence. Future research should therefore investigate whether coherence magnitude in frontal regions tracks with broader markers of severity, including impulsivity and comorbid affective symptoms.

Although interhemispheric coherence is susceptible to volume conduction, both GD and HCs groups were recorded under identical conditions, making group comparisons valid even if absolute values are affected. Interhemispheric coherence has long been used as a proxy for transcallosal communication and hemispheric integration in clinical and cognitive neuroscience (Nunez, 2000). Prior work has shown that IHC can distinguish between clinical and healthy populations in disorders such as depression (Knott et al., 2001), Alzheimer's disease (Sankari and Adeli, 2011), and aging (Duffy et al., 1996), as well as in broader psychiatric contexts (Markovska-Simoska et al., 2018). We therefore interpret IHC not as an absolute marker of true connectivity, but as a comparative measure highlighting functional differences between clinically distinct groups. This perspective situates our findings within a well-established tradition of EEG biomarker research while acknowledging the methodological constraints inherent to scalp EEG.

The hypoconnectivity we found in posterior regions, as anticipated in our hypothesis, also finds some support in the literature, albeit under certain conditions. For instance, Youh et al. (2017) studied EEG coherence in individuals with co-occurring “gaming disorder” and depression, and observed a marked decrease in interhemispheric frontal alpha coherence in the dual-diagnosis group compared to depressed patients without gaming addiction. That result (lower Fp1–Fp2 coherence) is in the opposite direction to our GD finding of higher Fp1–Fp2 coherence; however, it may be explained by differences in the sample (the presence of major depression in Youh's subjects could suppress frontal connectivity) or differences between video gaming and gambling behaviors. Notably, Youh et al. (2017) found that the reduced frontopolar coherence in their gaming-addicted group was linked to greater vulnerability to attentional problems, reinforcing the idea that insufficient frontal communication can impair cognitive control. In our non-depressed GD sample, frontal coherence was instead elevated, which might indicate a different pathophysiological profile when mood disorder is absent. Nonetheless, the concept of coherence aberrations relating to cognitive symptoms holds across studies: both decreased and increased coherence can be problematic if they reflect an imbalance in network integration. Additionally, our finding of parietal hypocoherence dovetails with the general notion that addiction entails dysfunctional attention networks. While few prior studies have examined parietal interhemispheric coherence in gambling, reduced beta-band coherence has been reported in other clinical contexts (e.g., in dementia and substance use disorder) and is often interpreted as a loss of efficient long-range coupling between cortical areas (Fide et al., 2023; Meyers et al., 2021). Consistent with this, individuals with GD show reductions in higher-frequency EEG activity (beta power) at baseline, and our results extend this to a connectivity dimension, suggesting that the parietal and central regions in gamblers may be less synchronized in the fast bands, potentially contributing to their known deficits in concentration and impulse regulation. Further evidence of disorder-specific profiles comes from Taigibova and Rabadanova (2022), who reported that Internet addicts showed decreased frontal coherence alongside heightened occipital synchrony, whereas game addicts demonstrated widespread frontal and temporal hypercoherence. These contrasting patterns suggest that although coherence abnormalities are common across behavioral addictions, their topography and frequency specificity may differ, potentially reflecting distinct compensatory network dynamics.

Overall, our findings align with and extend previous qEEG research by illustrating that GD is characterized by a coexistence of hypercoherent and hypocoherent networks, depending on brain region—an overall pattern consistent with our initial hypothesis. This dual configuration may reflect an imbalance between excessive coupling in frontal-limbic circuits (perhaps related to craving or compulsivity) and deficient connectivity in posterior cortical circuits (related to attention and cognitive control). Such an imbalance is in line with theoretical models of addiction neurocircuitry, which postulate overactive “drive”/reward networks and underactive control networks. From a clinical perspective, these coherence abnormalities hold promise as neurophysiological markers of GD. If reproducible, interhemispheric coherence measures could potentially aid in identifying individuals at risk for persistent gambling problems or serve as an objective outcome measure for interventions. For example, Park et al. (2018) conducted a longitudinal EEG study on patients with gaming disorder receiving therapy and found that pre-treatment elevations in beta coherence persisted even after symptomatic improvement, suggesting these connectivity traits might represent a stable vulnerability factor. It would be valuable to investigate whether similar coherence patterns in GD remain stable or normalize with successful treatment (e.g., cognitive-behavioral therapy or pharmacotherapy). Future research should also examine how coherence in specific bands relates to clinical features in GD—for instance, do gamblers with higher frontopolar coherence exhibit worse decision-making or greater craving? Conversely, might those with very low coherence in parietal regions have more pronounced attentional deficits or impulsivity? Addressing these questions could clarify the functional significance of the EEG coherence alterations we observed and further validate the hypothesized network imbalance as a core neurophysiological feature of GD.

## 5 Limitations

This study's retrospective, cross-sectional design and modest sample size limit the generalizability of the findings. The all-male sample prevents conclusions about potential sex differences in gambling-related connectivity patterns. As with all scalp EEG studies, coherence estimates can be influenced by factors such as volume conduction and reference choice, and these methodological constraints should be considered when interpreting the results. While interhemispheric analyses were prioritized to reduce the impact of short-range field spread, volume conduction cannot be fully eliminated, and this should be acknowledged as a limitation of scalp-level coherence. In addition, because of the modest sample size, we did not apply formal multiple-comparison correction or multivariate analyses such as ANOVA; instead, we relied on independent-samples *t*-tests and emphasized consistent regional patterns across frequency bands. This increases the risk of type I error, and future studies with larger samples should incorporate correction procedures or multivariate statistical models to strengthen inference. Another limitation is that a small number of GD participants presented with comorbid psychiatric disorders (e.g., ADHD, OCD). Although these cases were few, comorbidities may have contributed to variability in coherence patterns and represent a potential confounding factor. Furthermore, the cross-sectional nature of the data precludes causal inferences about whether the observed connectivity alterations represent predisposing traits, consequences of prolonged gambling behavior, or both. Future research should employ longitudinal designs, include more diverse samples, and incorporate methods that better minimize volume conduction effects and control for psychiatric comorbidities to clarify the temporal and causal nature of these coherence changes.

## 6 Conclusion

This study provides evidence, in line with our initial hypothesis, that resting-state interhemispheric EEG coherence is altered in GD, with increased coupling at frontal pole sites (Fp1–Fp2) and decreased coupling in temporal, central, and parietal regions across multiple frequency bands. These results suggest that GD involves a disruption of normal hemispheric communication, characterized by a hyper-integrated anterior network that may underlie maladaptive decision processes and hypo-integrated posterior networks that could contribute to lapses in attention and self-regulatory control. If replicated in larger and more diverse samples, these coherence patterns could serve as biomarkers for the neurophysiological state of GD and guide neuromodulatory interventions (for example, neurofeedback or brain stimulation) aimed at restoring network balance. Future research integrating coherence analysis with cognitive assessments and longitudinal designs will be essential to determine whether these EEG markers can track illness course or recovery. By situating our findings within the broader qEEG literature on addiction, we underscore that brain network coherence is a distinguishing feature of GD with potential to inform both the understanding of its neurobiology and the development of targeted treatments. We did not present intrahemispheric coherence results in detail, as they closely mirrored the interhemispheric findings and are more affected by volume conduction due to the short distances between adjacent electrodes. Future work using methods that minimize volume conduction (e.g., current source density or source-space coherence) could provide additional insight into intrahemispheric connectivity.

## Data Availability

The original contributions presented in the study are included in the article/Supplementary material, further inquiries can be directed to the corresponding author.
