# Supplementary material for: Interhemispheric EEG coherence as a candidate biomarker in gambling disorder: evidence of frontal hyperconnectivity and posterior disconnectivity
Source: Front Neurosci. 2025 Oct 24;19:1687112. doi: 10.3389/fnins.2025.1687112 (PMC12592092; doi:10.3389/fnins.2025.1687112)
Supplement: Supplementary file 1 [file Data_Sheet_1.docx]

**Interhemispheric qEEG Coherence as a Candidate Biomarker in Gambling Disorder**

**Rank raw p-values ​​from normal independent sampling from smallest to largest**

| **No** | **Band** | **Elektrot Couple** | **Raw p** |
| --- | --- | --- | --- |
| 1 | Alpha | C3-C4 | 0.001 |
| 2 | Alpha | P3-P4 | 0.006 |
| 3 | Delta | T5-T6 | 0.009 |
| 4 | Alpha | T3-T4 | 0.012 |
| 5 | Beta | C3-C4 | 0.001 |
| 6 | Beta | P3-P4 | 0.012 |
| 7 | Delta | FP1-FP2 | 0.015 |
| 8 | Delta | O1-O2 | 0.034 |
| 9 | Delta | T3-T4 | 0.031 |
| 10 | Theta | FP1-FP2 | 0.046 |
| 11 | Theta | P3-P4 | 0.046 |
| 12 | Beta | FP1-FP2 | 0.023 |
| 13 | Delta | F3-F4 | 0.064 |
| 14 | Delta | P3-P4 | 0.072 |
| 15 | Theta | T3-T4 | 0.054 |
| 16 | Theta | F3-F4 | 0.095 |
| 17 | Theta | T5-T6 | 0.096 |
| 18 | Delta | C3-C4 | 0.109 |
| 19 | Alpha | F3-F4 | 0.087 |
| 20 | Theta | C3-C4 | 0.225 |
| 21 | Beta | F3-F4 | 0.203 |
| 22 | Beta | F7-F8 | 0.212 |
| 23 | Theta | F7-F8 | 0.756 |
| 24 | Alpha | FP1-FP2 | 0.494 |
| 25 | Alpha | O1-O2 | 0.742 |
| 26 | Alpha | F7-F8 | 0.716 |
| 27 | Alpha | T5-T6 | 0.371 |
| 28 | Beta | O1-O2 | 0.418 |
| 29 | Beta | T3-T4 | 0.882 |
| 30 | Beta | T5-T6 | 0.607 |
| 31 | Delta | F7-F8 | 0.307 |
| 32 | Theta | O1-O2 | 0.971 |

**FDR Adjustment (Benjamini-Hochberg)**

To account for multiple comparisons, p-values from all 32 independent t-tests were ranked from smallest to largest. For each p-value, its rank (iii) and the total number of tests (m=32m = 32m=32) were used to calculate the FDR-adjusted p-value according to the formula:


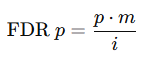


A result was considered significant if the FDR-adjusted p-value was ≤ 0.05.

**Example calculation:**

- The smallest p-value was 0.001 (Alpha band, C3-C4, rank i=1i = 1i=1):

FDR p=0.001×32/1=0.032 (significant)

- Another small p-value was 0.006 (Alpha band, P3-P4, rank i=2i = 2i=2):

FDR p=0.006×32/2=0.096 (not significant)

This procedure was applied to all p-values to determine which results remained significant after controlling for multiple comparisons.

| **Band** | **Elektrot** | **Ham p** | **FDR p** | **Significant?** |
| --- | --- | --- | --- | --- |
| **Alpha** | **C3-C4** | **0.001** | **0.032** | **Yes** |
| **Beta** | **C3-C4** | **0.001** | **0.032** | **Yes** |
| Delta | T5-T6 | 0.009 | 0.072 | No |
| Alpha | P3-P4 | 0.006 | 0.096 | No |
| Alpha | T3-T4 | 0.012 | 0.192 | No |
| Beta | P3-P4 | 0.012 | 0.192 | No |
| Delta | FP1-FP2 | 0.015 | 0.32 | No |
| Delta | O1-O2 | 0.034 | 0.272 | No |
| Delta | T3-T4 | 0.031 | 0.248 | No |
| Theta | FP1-FP2 | 0.046 | 0.307 | No |
| Theta | P3-P4 | 0.046 | 0.307 | No |
| Beta | FP1-FP2 | 0.023 | 0.32 | No |

To control the increased risk of false positives due to multiple comparisons, we applied the False Discovery Rate (FDR) correction using the Benjamini-Hochberg method. This method sorts all p-values ​​from smallest to largest and corrects p-values ​​by considering their ranking position and the total number of tests, thus controlling the expected false positive rate among results considered significant. Unlike more conservative methods that aim to completely suppress all effects, FDR allows the detection of true effects and limits the rate of false discoveries. In our dataset of 32 independent t-tests, this approach preserves the strongest effects while appropriately eliminating weak effects that lose significance after correction; differences are particularly pronounced between the C3-C4 electrode pairs in the Alpha and Beta bands.

**Significant after FDR: Alpha C3-C4 and Beta C3-C4**

**Significant after FDR: All other tests (Delta, Theta, other Alpha and Beta electrodes)**

**That is, the strongest effects are observed between the Alpha and Beta C3-C4 electrode pair; all other tests remain insignificant after FDR correction.**
