# Supplementary material for: Interhemispheric EEG coherence as a candidate biomarker in gambling disorder: evidence of frontal hyperconnectivity and posterior disconnectivity
Source: Front Neurosci. 2025 Oct 24;19:1687112. doi: 10.3389/fnins.2025.1687112 (PMC12592092; doi:10.3389/fnins.2025.1687112)
Supplement: Supplementary file 3 [file Data_Sheet_3.docx]

| *South Oaks Scale and Koheranslar Pearson's Correlations* -no correlation | | | | | | | | | |
| --- | --- | --- | --- | --- | --- | --- | --- | --- | --- |
|  | |  | |  | | Pearson's r | | p | |
| South_Oaks |  | - |  | Delta_FP1FP2 |  | 0.202 |  | 0.293 |  |
| South_Oaks |  | - |  | Delta_C3C4 |  | 0.075 |  | 0.700 |  |
| South_Oaks |  | - |  | Delta_O1O2 |  | 0.058 |  | 0.764 |  |
| South_Oaks |  | - |  | Delta_T3T4 |  | 0.011 |  | 0.954 |  |
| South_Oaks |  | - |  | Delta_F3F4 |  | 0.066 |  | 0.733 |  |
| South_Oaks |  | - |  | Delta_P3P4 |  | 0.051 |  | 0.795 |  |
| South_Oaks |  | - |  | Delta_F7F8 |  | -0.002 |  | 0.992 |  |
| South_Oaks |  | - |  | Delta_T5T6 |  | 0.011 |  | 0.954 |  |
| South_Oaks |  | - |  | Theta_FP1FP2 |  | 0.236 |  | 0.217 |  |
| South_Oaks |  | - |  | Theta_C3C4 |  | 0.227 |  | 0.237 |  |
| South_Oaks |  | - |  | Theta_O1O2 |  | -0.009 |  | 0.962 |  |
| South_Oaks |  | - |  | Theta_T3T4 |  | -0.023 |  | 0.906 |  |
| South_Oaks |  | - |  | Theta_F3F4 |  | 0.164 |  | 0.396 |  |
| South_Oaks |  | - |  | Theta_P3P4 |  | 0.098 |  | 0.613 |  |
| South_Oaks |  | - |  | Theta_F7F8 |  | 0.031 |  | 0.873 |  |
| South_Oaks |  | - |  | Theta_T5T6 |  | 0.093 |  | 0.630 |  |
| South_Oaks |  | - |  | Alpha_FP1FP2 |  | 0.292 |  | 0.124 |  |
| South_Oaks |  | - |  | Alpha_C3C4 |  | 0.148 |  | 0.445 |  |
| South_Oaks |  | - |  | Alpha_O1O2 |  | -0.116 |  | 0.549 |  |
| South_Oaks |  | - |  | Alpha_T3T4 |  | 0.063 |  | 0.745 |  |
| South_Oaks |  | - |  | Alpha_F3F4 |  | 0.139 |  | 0.473 |  |
| South_Oaks |  | - |  | Alpha_P3P4 |  | 0.030 |  | 0.877 |  |
| South_Oaks |  | - |  | Alpha_F7F8 |  | 0.150 |  | 0.436 |  |
| South_Oaks |  | - |  | Alpha_T5T6 |  | 0.158 |  | 0.412 |  |
| South_Oaks |  | - |  | Beta_FP1FP2 |  | 0.102 |  | 0.597 |  |
| South_Oaks |  | - |  | Beta_C3C4 |  | 0.002 |  | 0.993 |  |
| South_Oaks |  | - |  | Beta_O1O2 |  | -0.148 |  | 0.442 |  |
| South_Oaks |  | - |  | Beta_T3T4 |  | -0.106 |  | 0.584 |  |
| South_Oaks |  | - |  | Beta_F3F4 |  | 0.016 |  | 0.935 |  |
| South_Oaks |  | - |  | Beta_P3P4 |  | 0.012 |  | 0.951 |  |
| South_Oaks |  | - |  | Beta_F7F8 |  | -0.177 |  | 0.359 |  |
| South_Oaks |  | - |  | Beta_T5T6 |  | -0.136 |  | 0.482 |  |
|  | | | | | | | | | |
| * p < .05, ** p < .01, *** p < .001 | | | | | | | | | |

| *Year of Dirsorders and Coherence correlations were performed using Pearson's Partial Correlations after holding age constant - significant correlations exist, both positive and negative* | | | | | | | | | |
| --- | --- | --- | --- | --- | --- | --- | --- | --- | --- |
|  | |  | |  | | Pearson's r | | p | |
| Disorders_Year |  | - |  | Delta_FP1FP2 |  | 0.106 |  | 0.593 |  |
| Disorders _Year |  | - |  | Delta_C3C4 |  | 0.281 |  | 0.148 |  |
| Disorders _Year |  | - |  | Delta_O1O2 |  | 0.402 | * | 0.034 |  |
|  |  | - |  | Delta_T3T4 |  | -0.067 |  | 0.733 |  |
|  |  | - |  | Delta_F3F4 |  | 0.157 |  | 0.426 |  |
|  |  | - |  | Delta_P3P4 |  | 0.295 |  | 0.127 |  |
|  |  | - |  | Delta_F7F8 |  | -0.197 |  | 0.315 |  |
|  |  | - |  | Delta_T5T6 |  | 0.264 |  | 0.175 |  |
|  |  | - |  | Theta_FP1FP2 |  | -0.189 |  | 0.336 |  |
|  |  | - |  | Theta_C3C4 |  | 0.054 |  | 0.787 |  |
|  |  | - |  | Theta_O1O2 |  | 0.311 |  | 0.107 |  |
|  |  | - |  | Theta_T3T4 |  | -0.211 |  | 0.280 |  |
|  |  | - |  | Theta_F3F4 |  | -0.220 |  | 0.261 |  |
|  |  | - |  | Theta_P3P4 |  | 0.220 |  | 0.260 |  |
|  |  | - |  | Theta_F7F8 |  | -0.346 |  | 0.071 |  |
|  |  | - |  | Theta_T5T6 |  | 0.315 |  | 0.103 |  |
|  |  | - |  | Alpha_FP1FP2 |  | -0.284 |  | 0.144 |  |
|  |  | - |  | Alpha_C3C4 |  | 0.075 |  | 0.706 |  |
|  |  | - |  | Alpha_O1O2 |  | 0.343 |  | 0.074 |  |
|  |  | - |  | Alpha_T3T4 |  | 0.223 |  | 0.253 |  |
|  |  | - |  | Alpha_F3F4 |  | -0.127 |  | 0.521 |  |
|  |  | - |  | Alpha_P3P4 |  | 0.202 |  | 0.302 |  |
|  |  | - |  | Alpha_F7F8 |  | -0.111 |  | 0.573 |  |
|  |  | - |  | Alpha_T5T6 |  | 0.308 |  | 0.111 |  |
| Disorders _Year |  | - |  | Beta_FP1FP2 |  | -0.388 | * | 0.041 |  |
|  |  | - |  | Beta_C3C4 |  | 0.042 |  | 0.830 |  |
|  |  | - |  | Beta_O1O2 |  | 0.188 |  | 0.339 |  |
|  |  | - |  | Beta_T3T4 |  | 0.067 |  | 0.735 |  |
| Disorders _Year |  | - |  | Beta_F3F4 |  | -0.513 | ** | 0.005 |  |
|  |  | - |  | Beta_P3P4 |  | 0.063 |  | 0.751 |  |
|  |  | - |  | Beta_F7F8 |  | -0.388 | * | 0.041 |  |
|  |  | - |  | Beta_T5T6 |  | 0.081 |  | 0.682 |  |
|  | | | | | | | | | |
| *Note.*  Conditioned on variables: Age. | | | | | | | | | |
| * p < .05, ** p < .01, *** p < .001 | | | | | | | | | |

#### Disorder_Year vs. Delta_O1O2


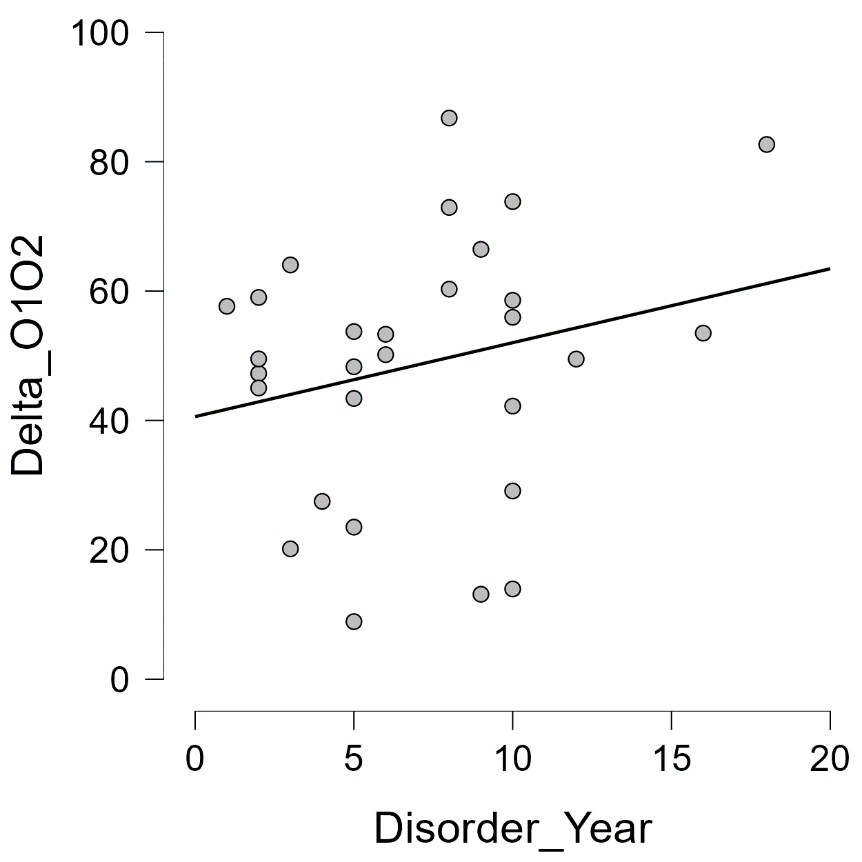


#### Disorder_Year vs. Beta_FP1FP2


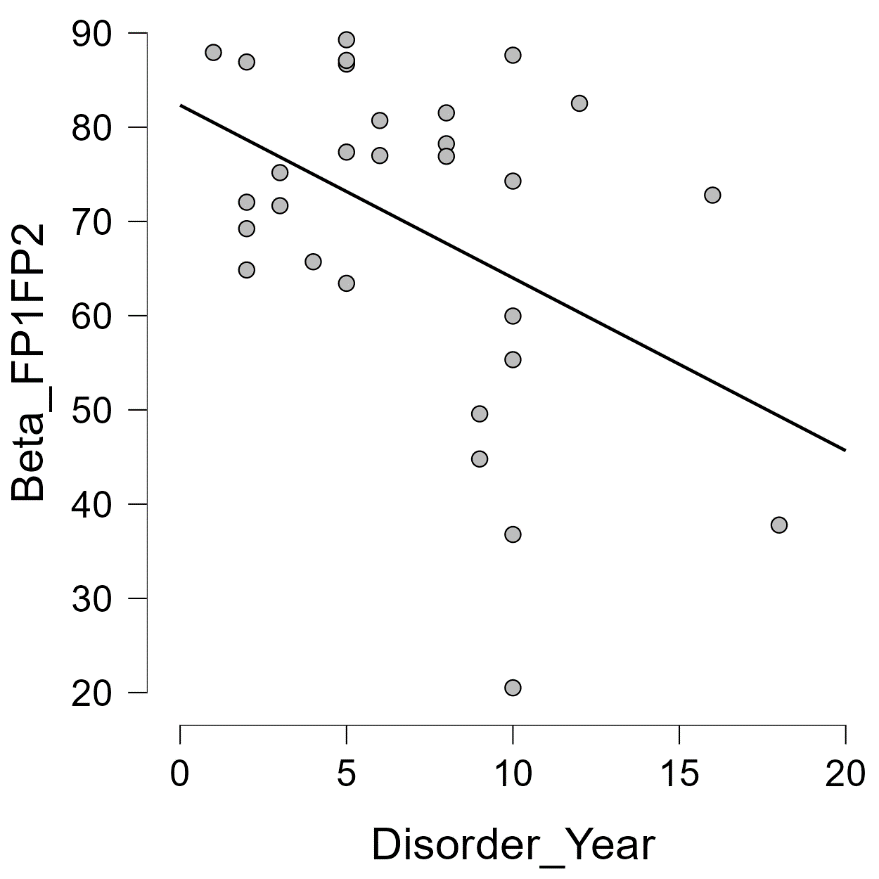


#### Disorder_Year vs. Beta_F3F4


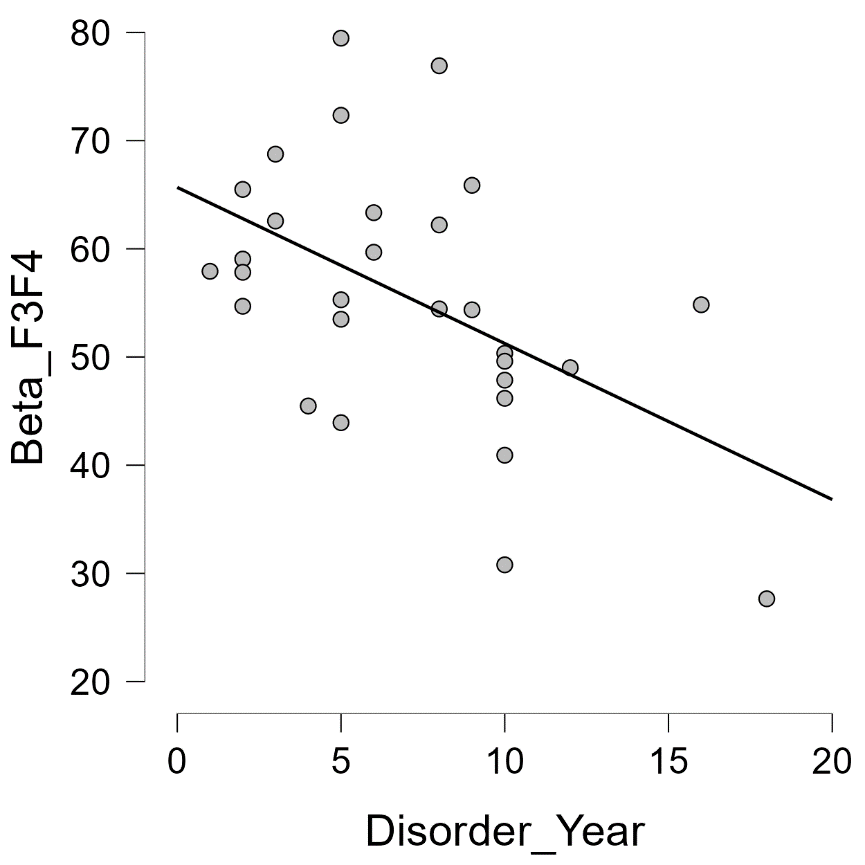


**Correlation**

| *Pearson's Correlations* | | | | | | | | | |
| --- | --- | --- | --- | --- | --- | --- | --- | --- | --- |
|  | |  | |  | | Pearson's r | | p | |
| Beck_anxiety |  | - |  | Beck_depression |  | 0.646 | *** | < .001 |  |
| Beck_anxiety |  | - |  | South_Oaks |  | 0.226 |  | 0.238 |  |
| Beck_anxiety |  | - |  | Addiction_Year |  | -0.090 |  | 0.643 |  |
| Beck_depression |  | - |  | South_Oaks |  | 0.247 |  | 0.197 |  |
| Beck_depression |  | - |  | Addiction_Year |  | -0.180 |  | 0.351 |  |
| South_Oaks |  | - |  | Addiction_Year |  | 0.005 |  | 0.980 |  |
|  | | | | | | | | | |
| * p < .05, ** p < .01, *** p < .001 | | | | | | | | | |
