# Supplementary material for: Interhemispheric EEG coherence as a candidate biomarker in gambling disorder: evidence of frontal hyperconnectivity and posterior disconnectivity
Source: Front Neurosci. 2025 Oct 24;19:1687112. doi: 10.3389/fnins.2025.1687112 (PMC12592092; doi:10.3389/fnins.2025.1687112)
Supplement: Supplementary file 4 [file Data_Sheet_4.doc]

Group = Healthy Control Age

Statistics	
Age  	
N	Valid	45	
	Missing	0	
Mean	35.13	
Median	36.00	
Std. Deviation	8.704	
Range	31	
Minimum	18	
Maximum	49	
a. Group = Healthy Control	


Group = Gambling disorder Age

Statistics	
Age  	
N	Valid	29	
	Missing	0	
Mean	32.86	
Median	31.00	
Std. Deviation	10.109	
Range	43	
Minimum	18	
Maximum	61	
a. Group = Gambling disorder	


Group = Gambling Disorderda Komorbid durumlar 
Komorbidite	
	Frequency	Percent	Valid Percent	Cumulative Percent	
Valid		25	86.2	86.2	86.2	
	DEHB	3	10.3	10.3	96.6	
	OKB	1	3.4	3.4	100.0	
	Total	29	100.0	100.0		
a. Group = Gambling disorder	


Group = Gambling disorder
Education	
	Frequency	Percent	Valid Percent	Cumulative Percent	
Valid	Ýlkokul	2	6.9	6.9	6.9	
	Lisans	6	20.7	20.7	27.6	
	Lise	16	55.2	55.2	82.8	
	Önlisans	1	3.4	3.4	86.2	
	Ortaokul	1	3.4	3.4	89.7	
	Üniversite	2	6.9	6.9	96.6	
	Yüksek Lisans	1	3.4	3.4	100.0	
	Total	29	100.0	100.0		
a. Group = Gambling disorder	


Group = Gambling disorder çocuk sahibi olma durumu ve sayýsý

Child	
	Frequency	Percent	Valid Percent	Cumulative Percent	
Valid	0	14	48.3	48.3	48.3	
	1	10	34.5	34.5	82.8	
	2	3	10.3	10.3	93.1	
	3	2	6.9	6.9	100.0	
	Total	29	100.0	100.0		
a. Group = Gambling disorder	


Gambling Disorders Year

Statistics	
Disorders_Year  	
N	Valid	29	
	Missing	0	
Mean	7.03	
Median	6.00	
Std. Deviation	4.187	
Range	17	
Minimum	1	
Maximum	18	
a. Group = Gambling disorder	
