# Supplementary material for: Interhemispheric EEG coherence as a candidate biomarker in gambling disorder: evidence of frontal hyperconnectivity and posterior disconnectivity
Source: Front Neurosci. 2025 Oct 24;19:1687112. doi: 10.3389/fnins.2025.1687112 (PMC12592092; doi:10.3389/fnins.2025.1687112)
Supplement: Supplementary file 5 [file Data_Sheet_5.doc]

GET
  FILE='D:\2025 Çalýþma Grubumuz\Kumar Baðýmlýlýðý Koherans\00 KOHERANS Age Matched 45 HCs ve 29 Kumar Bozukluðu.sav'.
DATASET NAME $DataSet WINDOW=FRONT.
T-TEST GROUPS=Group(1 0)
  /MISSING=ANALYSIS
  /VARIABLES=Delta_FP1FP2 Delta_C3C4 Delta_O1O2 Delta_T3T4 Delta_F3F4 Delta_P3P4 Delta_F7F8
    Delta_T5T6 Theta_FP1FP2 Theta_C3C4 Theta_O1O2 Theta_T3T4 Theta_F3F4 Theta_P3P4 Theta_F7F8
    Theta_T5T6 Alpha_FP1FP2 Alpha_C3C4 Alpha_O1O2 Alpha_T3T4 Alpha_F3F4 Alpha_P3P4 Alpha_F7F8
    Alpha_T5T6 Beta_FP1FP2 Beta_C3C4 Beta_O1O2 Beta_T3T4 Beta_F3F4 Beta_P3P4 Beta_F7F8 Beta_T5T6
  /ES DISPLAY(TRUE)
  /CRITERIA=CI(.95).


T-Test

[$DataSet] D:\2025 Çalýþma Grubumuz\Kumar Baðýmlýlýðý Koherans\00 KOHERANS Age Matched 45 HCs ve 29 Kumar Bozukluðu.sav

Group Statistics	
	Group	N	Mean	Std. Deviation	Std. Error Mean	
Delta_FP1 FP2	Gambling disorder	29	67.4131	23.57548	4.37786	
	Healthy Control	45	53.1344	24.38594	3.63524	
Delta_C3 C4	Gambling disorder	29	52.6552	22.87321	4.24745	
	Healthy Control	45	60.6907	19.31175	2.87883	
Delta_O1 O2	Gambling disorder	29	48.6314	20.12519	3.73715	
	Healthy Control	45	59.0351	20.17989	3.00824	
Delta_T3 T4	Gambling disorder	29	10.9017	11.22656	2.08472	
	Healthy Control	45	17.2380	12.65411	1.88636	
Delta_F3 F4	Gambling disorder	29	58.7917	19.23036	3.57099	
	Healthy Control	45	66.2927	14.93434	2.22628	
Delta_P3 P4	Gambling disorder	29	52.6786	23.05439	4.28109	
	Healthy Control	45	62.1071	20.81207	3.10248	
Delta_F7 F8	Gambling disorder	29	22.7831	17.44830	3.24007	
	Healthy Control	45	27.0611	17.47961	2.60571	
Delta_T5 T6	Gambling disorder	29	22.9621	16.21087	3.01028	
	Healthy Control	45	33.4491	16.31725	2.43243	
Theta_FP1 FP2	Gambling disorder	29	73.1841	18.07028	3.35557	
	Healthy Control	45	63.9596	19.69862	2.93650	
Theta_C3 C4	Gambling disorder	29	53.7966	16.08110	2.98619	
	Healthy Control	45	58.7253	17.43110	2.59848	
Theta_O1 O2	Gambling disorder	29	54.8686	16.77959	3.11589	
	Healthy Control	45	55.0204	17.73555	2.64386	
Theta_T3 T4	Gambling disorder	29	5.5703	6.67655	1.23980	
	Healthy Control	45	9.2613	8.60138	1.28222	
Theta_F3 F4	Gambling disorder	29	63.6197	13.72117	2.54796	
	Healthy Control	45	68.6069	11.41419	1.70153	
Theta_P3 P4	Gambling disorder	29	51.3748	17.57617	3.26381	
	Healthy Control	45	59.8973	17.67304	2.63454	
Theta_F7 F8	Gambling disorder	29	23.6221	16.54394	3.07213	
	Healthy Control	45	24.7371	13.96989	2.08251	
Theta_T5 T6	Gambling disorder	29	15.2672	11.71177	2.17482	
	Healthy Control	45	20.0629	12.07400	1.79989	
Alpha_FP1 FP2	Gambling disorder	29	85.3648	13.76658	2.55639	
	Healthy Control	45	83.1840	13.00559	1.93876	
Alpha_C3 C4	Gambling disorder	29	49.6010	16.80725	3.12103	
	Healthy Control	45	63.1827	16.59979	2.47455	
Alpha_O1 O2	Gambling disorder	29	61.5710	17.09049	3.17362	
	Healthy Control	45	60.1987	17.71137	2.64025	
Alpha_T3 T4	Gambling disorder	29	5.4752	5.56476	1.03335	
	Healthy Control	45	12.5107	13.88022	2.06914	
Alpha_F3 F4	Gambling disorder	29	76.1786	13.62824	2.53070	
	Healthy Control	45	80.8827	9.68169	1.44326	
Alpha_P3 P4	Gambling disorder	29	49.5921	18.35365	3.40819	
	Healthy Control	45	60.6860	15.17049	2.26148	
Alpha_F7 F8	Gambling disorder	29	43.1366	23.02327	4.27531	
	Healthy Control	45	44.9309	18.91571	2.81979	
Alpha_T5 T6	Gambling disorder	29	20.7590	15.92702	2.95757	
	Healthy Control	45	24.1242	15.56960	2.32098	
Beta_FP1 FP2	Gambling disorder	29	69.4393	17.50874	3.25129	
	Healthy Control	45	59.0942	19.44586	2.89882	
Beta_C3 C4	Gambling disorder	29	36.2183	11.44793	2.12583	
	Healthy Control	45	47.1804	12.07765	1.80043	
Beta_O1 O2	Gambling disorder	29	51.7579	13.02667	2.41899	
	Healthy Control	45	49.3576	11.92384	1.77750	
Beta_T3 T4	Gambling disorder	29	3.3900	5.94260	1.10351	
	Healthy Control	45	3.6227	6.95936	1.03744	
Beta_F3 F4	Gambling disorder	29	55.5276	11.93316	2.21593	
	Healthy Control	45	59.3618	12.91266	1.92491	
Beta_P3 P4	Gambling disorder	29	39.6655	14.81989	2.75198	
	Healthy Control	45	48.0560	12.96719	1.93303	
Beta_F7 F8	Gambling disorder	29	17.3041	13.76688	2.55645	
	Healthy Control	45	20.9689	11.14455	1.66133	
Beta_T5 T6	Gambling disorder	29	9.5976	7.82473	1.45302	
	Healthy Control	45	8.6547	7.56264	1.12737	


Independent Samples Test	
	Levene's Test for Equality of Variances	t-test for Equality of Means	
	F	Sig.	t	df	Sig. (2-tailed)	Mean Difference	95% Confidence Interval of the Difference	
							Lower	Upper	
Delta_FP1 FP2	Equal variances assumed	0.155	0.695	2.491	72	0.015	14.27866	2.85074	25.70658	
	Equal variances not assumed			2.509	61.360	0.015	14.27866	2.90136	25.65596	
Delta_C3 C4	Equal variances assumed	2.168	0.145	-1.625	72	0.109	-8.03549	-17.89475	1.82376	
	Equal variances not assumed			-1.566	52.574	0.123	-8.03549	-18.32918	2.25819	
Delta_O1 O2	Equal variances assumed	0.014	0.907	-2.167	72	0.034	-10.40373	-19.97303	-0.83443	
	Equal variances not assumed			-2.169	60.008	0.034	-10.40373	-20.00010	-0.80737	
Delta_T3 T4	Equal variances assumed	0.141	0.708	-2.196	72	0.031	-6.33628	-12.08914	-0.58341	
	Equal variances not assumed			-2.254	64.924	0.028	-6.33628	-11.95131	-0.72124	
Delta_F3 F4	Equal variances assumed	4.109	0.046	-1.882	72	0.064	-7.50094	-15.44578	0.44390	
	Equal variances not assumed			-1.782	49.260	0.081	-7.50094	-15.95635	0.95446	
Delta_P3 P4	Equal variances assumed	0.380	0.540	-1.824	72	0.072	-9.42849	-19.73499	0.87801	
	Equal variances not assumed			-1.783	55.408	0.080	-9.42849	-20.02227	1.16529	
Delta_F7 F8	Equal variances assumed	0.075	0.784	-1.028	72	0.307	-4.27801	-12.56979	4.01378	
	Equal variances not assumed			-1.029	59.968	0.308	-4.27801	-12.59504	4.03903	
Delta_T5 T6	Equal variances assumed	0.006	0.939	-2.706	72	0.009	-10.48704	-18.21324	-2.76085	
	Equal variances not assumed			-2.710	60.176	0.009	-10.48704	-18.22815	-2.74593	
Theta_FP1 FP2	Equal variances assumed	0.019	0.890	2.030	72	0.046	9.22458	0.16641	18.28275	
	Equal variances not assumed			2.069	63.579	0.043	9.22458	0.31553	18.13363	
Theta_C3 C4	Equal variances assumed	0.100	0.753	-1.223	72	0.225	-4.92878	-12.96018	3.10262	
	Equal variances not assumed			-1.245	63.345	0.218	-4.92878	-12.83828	2.98072	
Theta_O1 O2	Equal variances assumed	0.107	0.745	-0.037	72	0.971	-0.15182	-8.39737	8.09373	
	Equal variances not assumed			-0.037	62.286	0.970	-0.15182	-8.31970	8.01605	
Theta_T3 T4	Equal variances assumed	3.913	0.052	-1.960	72	0.054	-3.69099	-7.44525	0.06327	
	Equal variances not assumed			-2.069	69.403	0.042	-3.69099	-7.24879	-0.13319	
Theta_F3 F4	Equal variances assumed	3.779	0.056	-1.694	72	0.095	-4.98723	-10.85576	0.88129	
	Equal variances not assumed			-1.628	51.966	0.110	-4.98723	-11.13543	1.16096	
Theta_P3 P4	Equal variances assumed	0.001	0.982	-2.029	72	0.046	-8.52251	-16.89404	-0.15097	
	Equal variances not assumed			-2.032	60.130	0.047	-8.52251	-16.91225	-0.13276	
Theta_F7 F8	Equal variances assumed	1.707	0.195	-0.312	72	0.756	-1.11504	-8.24665	6.01656	
	Equal variances not assumed			-0.300	52.580	0.765	-1.11504	-8.56065	6.33057	
Theta_T5 T6	Equal variances assumed	0.018	0.892	-1.687	72	0.096	-4.79565	-10.46092	0.86963	
	Equal variances not assumed			-1.699	61.216	0.094	-4.79565	-10.44022	0.84893	
Alpha_FP1 FP2	Equal variances assumed	0.560	0.457	0.688	72	0.494	2.18083	-4.13586	8.49751	
	Equal variances not assumed			0.680	57.390	0.499	2.18083	-4.24296	8.60462	
Alpha_C3 C4	Equal variances assumed	0.102	0.750	-3.419	72	0.001	-13.58163	-21.49999	-5.66327	
	Equal variances not assumed			-3.410	59.345	0.001	-13.58163	-21.55061	-5.61265	
Alpha_O1 O2	Equal variances assumed	0.011	0.918	0.330	72	0.742	1.37237	-6.92184	9.66657	
	Equal variances not assumed			0.332	61.442	0.741	1.37237	-6.88147	9.62621	
Alpha_T3 T4	Equal variances assumed	18.126	0.000	-2.593	72	0.012	-7.03549	-12.44331	-1.62768	
	Equal variances not assumed			-3.042	62.569	0.003	-7.03549	-11.65793	-2.41306	
Alpha_F3 F4	Equal variances assumed	5.655	0.020	-1.736	72	0.087	-4.70405	-10.10626	0.69816	
	Equal variances not assumed			-1.615	46.074	0.113	-4.70405	-10.56801	1.15992	
Alpha_P3 P4	Equal variances assumed	1.984	0.163	-2.827	72	0.006	-11.09393	-18.91774	-3.27012	
	Equal variances not assumed			-2.712	51.706	0.009	-11.09393	-19.30271	-2.88516	
Alpha_F7 F8	Equal variances assumed	1.889	0.174	-0.366	72	0.716	-1.79434	-11.57818	7.98950	
	Equal variances not assumed			-0.350	51.462	0.728	-1.79434	-12.07389	8.48521	
Alpha_T5 T6	Equal variances assumed	0.006	0.938	-0.900	72	0.371	-3.36526	-10.82258	4.09207	
	Equal variances not assumed			-0.895	58.893	0.374	-3.36526	-10.88837	4.15786	
Beta_FP1 FP2	Equal variances assumed	0.333	0.566	2.321	72	0.023	10.34509	1.46043	19.22974	
	Equal variances not assumed			2.375	64.338	0.021	10.34509	1.64402	19.04616	
Beta_C3 C4	Equal variances assumed	2.461	0.121	-3.889	72	0.001	-10.96217	-16.58107	-5.34327	
	Equal variances not assumed			-3.935	62.207	0.000	-10.96217	-16.53054	-5.39380	
Beta_O1 O2	Equal variances assumed	0.203	0.654	0.815	72	0.418	2.40038	-3.46901	8.26976	
	Equal variances not assumed			0.800	56.009	0.427	2.40038	-3.61301	8.41376	
Beta_T3 T4	Equal variances assumed	0.273	0.603	-0.148	72	0.882	-0.23267	-3.35744	2.89211	
	Equal variances not assumed			-0.154	66.373	0.878	-0.23267	-3.25635	2.79102	
Beta_F3 F4	Equal variances assumed	0.005	0.944	-1.284	72	0.203	-3.83419	-9.78732	2.11894	
	Equal variances not assumed			-1.306	63.273	0.196	-3.83419	-9.69930	2.03091	
Beta_P3 P4	Equal variances assumed	0.247	0.621	-2.569	72	0.012	-8.39048	-14.90215	-1.87882	
	Equal variances not assumed			-2.495	54.070	0.016	-8.39048	-15.13277	-1.64819	
Beta_F7 F8	Equal variances assumed	0.480	0.491	-1.258	72	0.212	-3.66475	-9.47096	2.14146	
	Equal variances not assumed			-1.202	50.870	0.235	-3.66475	-9.78594	2.45643	
Beta_T5 T6	Equal variances assumed	0.003	0.956	0.517	72	0.607	0.94292	-2.69595	4.58179	
	Equal variances not assumed			0.513	58.393	0.610	0.94292	-2.73787	4.62371	

Independent Samples Effect Sizes	
	Effect Size	95% Confidence Interval	
		Lower	Upper	
Delta_FP1 FP2	Cohen's d	0.593	0.115	1.068	
Delta_C3 C4	Cohen's d	-0.387	-0.857	0.085	
Delta_O1 O2	Cohen's d	-0.516	-0.989	-0.040	
Delta_T3 T4	Cohen's d	-0.523	-0.996	-0.047	
Delta_F3 F4	Cohen's d	-0.448	-0.919	0.026	
Delta_P3 P4	Cohen's d	-0.434	-0.905	0.039	
Delta_F7 F8	Cohen's d	-0.245	-0.712	0.224	
Delta_T5 T6	Cohen's d	-0.644	-1.121	-0.164	
Theta_FP1 FP2	Cohen's d	0.483	0.008	0.955	
Theta_C3 C4	Cohen's d	-0.291	-0.759	0.179	
Theta_O1 O2	Cohen's d	-0.009	-0.475	0.458	
Theta_T3 T4	Cohen's d	-0.467	-0.938	0.008	
Theta_F3 F4	Cohen's d	-0.403	-0.873	0.069	
Theta_P3 P4	Cohen's d	-0.483	-0.955	-0.008	
Theta_F7 F8	Cohen's d	-0.074	-0.541	0.393	
Theta_T5 T6	Cohen's d	-0.402	-0.872	0.071	
Alpha_FP1 FP2	Cohen's d	0.164	-0.304	0.631	
Alpha_C3 C4	Cohen's d	-0.814	-1.297	-0.326	
Alpha_O1 O2	Cohen's d	0.079	-0.389	0.545	
Alpha_T3 T4	Cohen's d	-0.618	-1.093	-0.138	
Alpha_F3 F4	Cohen's d	-0.413	-0.884	0.060	
Alpha_P3 P4	Cohen's d	-0.673	-1.150	-0.191	
Alpha_F7 F8	Cohen's d	-0.087	-0.554	0.380	
Alpha_T5 T6	Cohen's d	-0.214	-0.682	0.255	
Beta_FP1 FP2	Cohen's d	0.553	0.076	1.026	
Beta_C3 C4	Cohen's d	-0.926	-1.414	-0.433	
Beta_O1 O2	Cohen's d	0.194	-0.274	0.661	
Beta_T3 T4	Cohen's d	-0.035	-0.502	0.432	
Beta_F3 F4	Cohen's d	-0.306	-0.774	0.165	
Beta_P3 P4	Cohen's d	-0.612	-1.087	-0.132	
Beta_F7 F8	Cohen's d	-0.300	-0.768	0.171	
Beta_T5 T6	Cohen's d	0.123	-0.345	0.590	
